# Supplementary material for: ZNF598 Is a Quality Control Sensor of Collided Ribosomes
Source: Mol Cell. 2018 Nov 1;72(3):469–481.e7. doi: 10.1016/j.molcel.2018.08.037 (PMC6224477; doi:10.1016/j.molcel.2018.08.037)
Supplement: Document S1. Figures S1–S7 and Table S1 [file mmc1.pdf]

Molecular Cell, Volume *72*

Supplemental Information

ZNF598 Is a Quality Control Sensor of Collided Ribosomes

Szymon Juszkiewicz, Viswanathan Chandrasekaran, Zhewang Lin, Sebastian Kraatz, V. Ramakrishnan, and Ramanujan S. Hegde

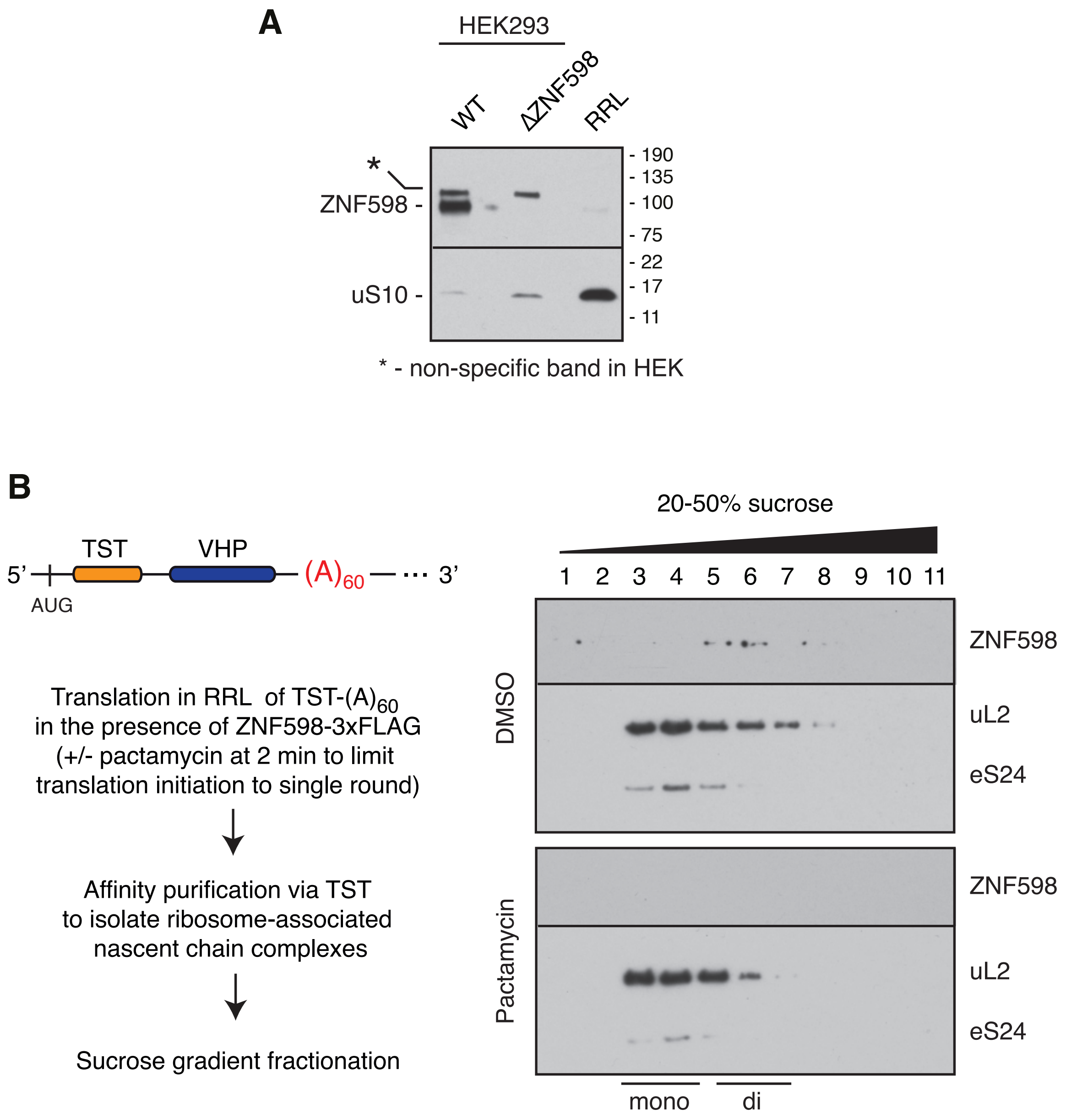

Fig. S1. ZNF598 engages a subpopulation of poly(A)-stalled ribosomes in vitro (related to Fig. 1)
(A) Lysates from WT or ΔZNF598 HEK293 cells were compared with rabbit reticulocyte lysate (RRL) by immunoblotting. Only a trace amount of ZNF598 was detected in RRL despite over-loading the sample (as judged by ribosomal protein uL10 levels). **(B)** Left panel: schematic representation of the TST-$(A)_{60}$ construct and experimental strategy to confine initiation in vitro to a single round by adding an initiation inhibitor (pactamycin) 2 min after the start of translation. Right panel: ribosome-nascent chain complexes from the mock treated (top) or pactamycin treated (bottom) translation reactions of TST-$(A)_{60}$ were affinity purified via the nascent chain. Equal amounts of recovered ribosomal complexes (as judged by absorbance at 260 nm) were separated by centrifugation through a sucrose gradient followed by immunoblotting of individual fractions for ZNF598 and ribosomal proteins. The positions of mono-ribosome and di-ribosome fractions are indicated. Note that recovery of ZNF598 with the ribosomal complexes is diminished when re-initiation is minimized. The reduced proportion of ribosomes in the di-ribosome fractions of the pactamycin-treated sample verifies that re-initiation was indeed reduced.

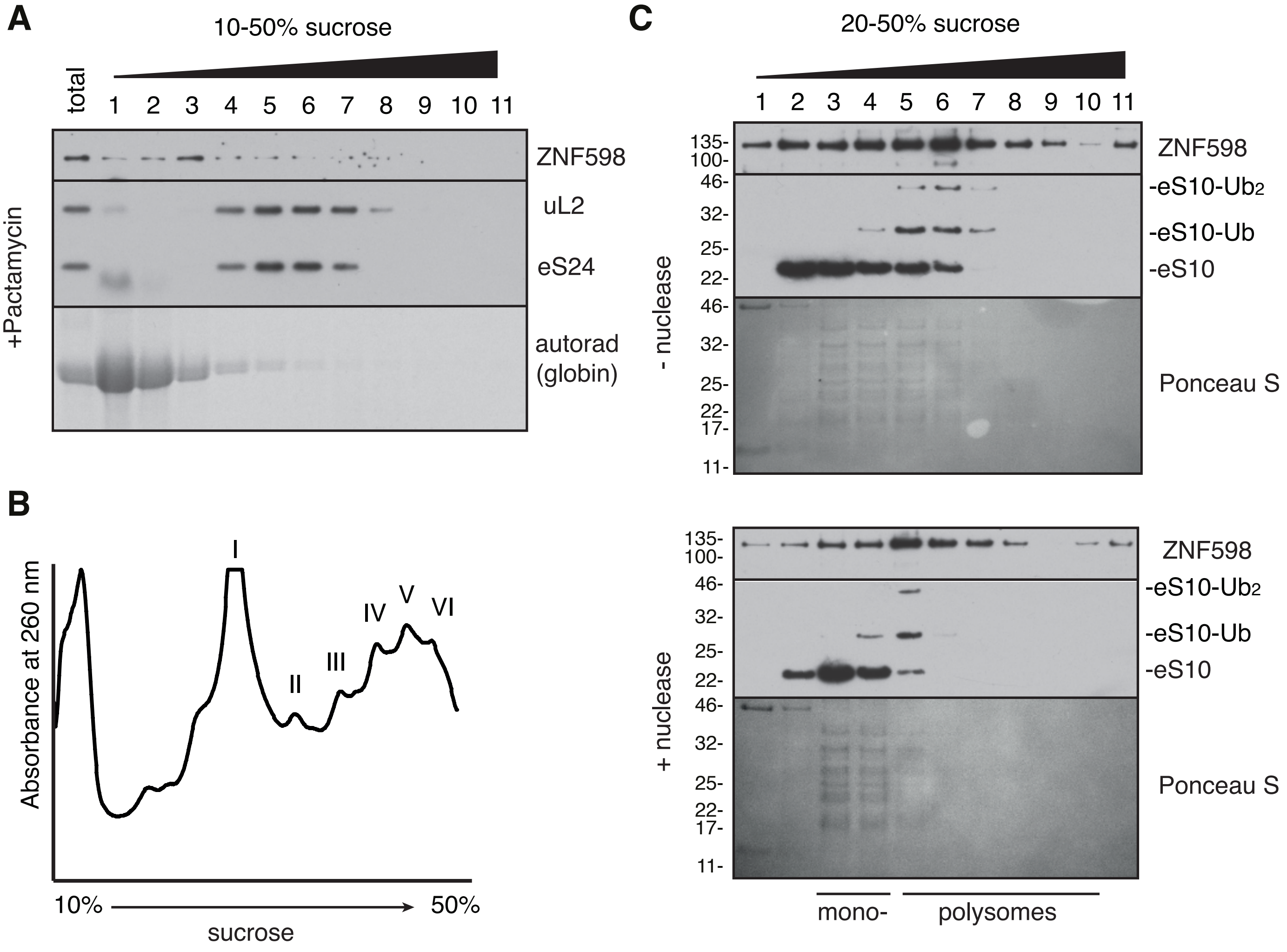

Fig. S2. Site-specific stalling of native polysomes triggers ZNF598 engagement (Related to Fig. 2)
(A) Native RRL was supplemented with 5 nM ZNF598 and the initiation inhibitor pactamycin before allowing ribosomes to run-off in the presence of ^{35}S-methionine. The reaction was analzyed on a sucrose gradient followed by autoradiography (to detect newly translated globin) or immunblotting for ZNF598 and ribosomal proteins. This reaction was performed and analyzed in parallel with that shown in Fig. 2B, which was stalled at the termination codon with eRF1^{AAQ}. Comparison of the two results indicates that the run-off sample produces full length globin that is not ribosome-associated, while the eRF1^{AAQ}-stalled sample contains an appreciable proportion of ribosome-associated globin. Furthermore, ZNF598 in the run-off sample is not stably associated with ribosomes, in contrast to the eRF1^{AAQ}-stalled sample. **(B)** Native RRL supplemented with eRF1^{AAQ} was allowed to translate for 10 min as in Fig. 2B before separation by sucrose gradient. The absorbance at 260 nm across the gradient is shown. Peak fractions corresponding to monosomes through pentasomes (I through V, respectively) were recovered for in vitro ubiquitination reactions with purified ZNF598 as shown on Figure 2D. The tetrasome fraction was also the sample used for subsequent structural analysis. **(C)** An experiment as Figure 2E is shown with the ubiquitinated and unmodified forms of eS10 detected simultaneously on the same strip of the blot. Note that after nuclease digestion, eS10 in the nuclease-resistant polysome fraction (lane 5) is almost completely ubiquitinated, whereas eS10 in the monosome fractions (lane 3) is almost entirely unmodified. ZNF598 is seen across the gradient due to over-exposure of the blot and some dissociation during the gradient centrifugation. Nevertheless, it is enriched in the fractions that also show the most eS10 ubiquitination.

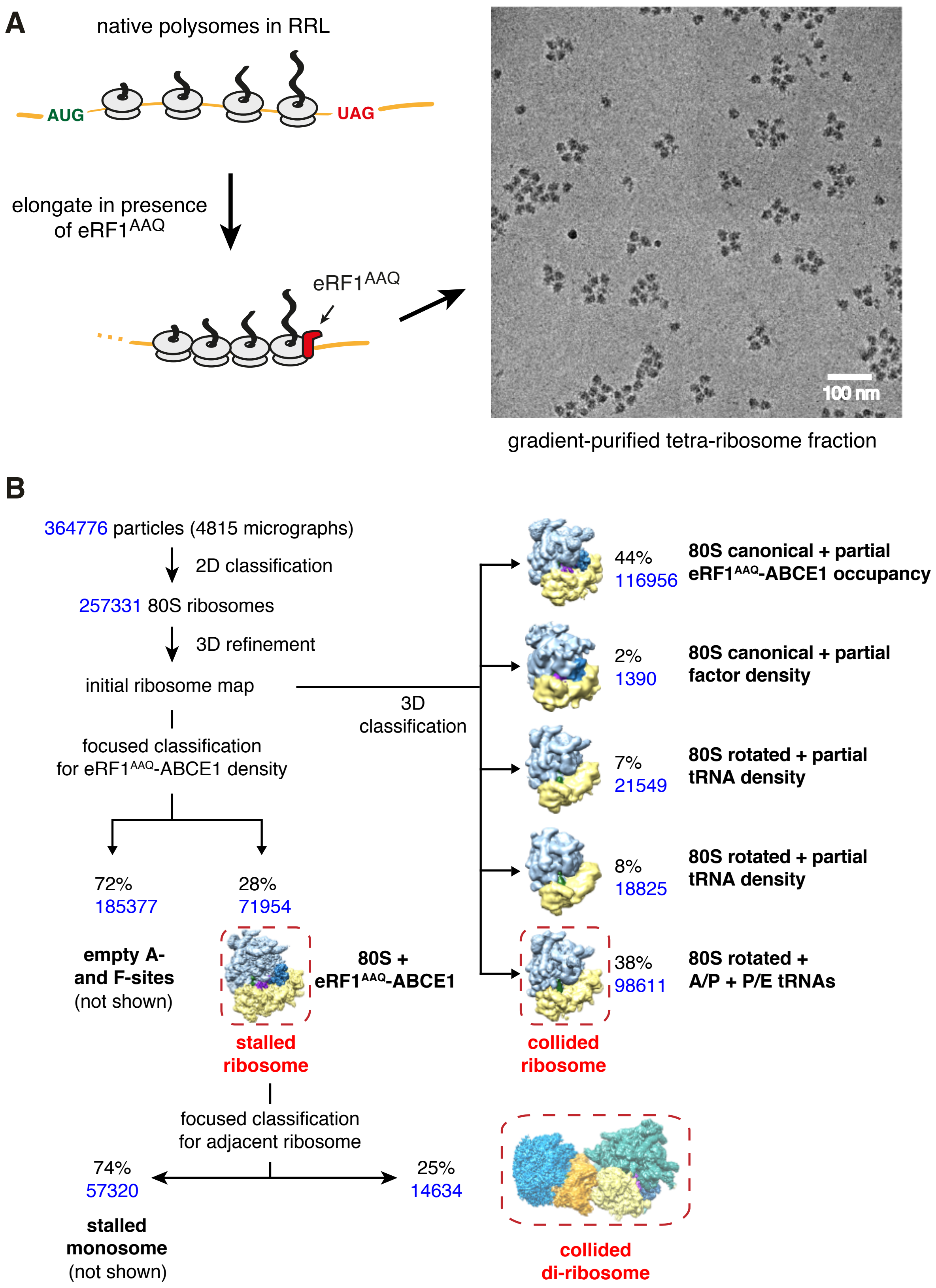

Fig. S3. Experimental strategy and classification scheme for structure determination (related to Fig. 3) **(A)** Schematic for generating native (primarily globin) stalled poly-ribosome complexes for cryo-EM analysis. Experimental details are provided in the Methods. The micrograph shows that although the tetra-ribosome fraction was analyzed, contamination with tri- and penta-ribosomes is readily seen. In addition, some disassembly during vitrification results in mono-ribosomes. **(B)** Overview of the classification scheme used to identify the leading stalled ribosomes and trailing collided ribosomes. Particles representing the stalled ribosome were used for additional classification to identify those that contain an adjacent collided ribosome, yielding a collided di-ribosome map. The particles represented in the red boxes were subjected to movie processing, particle polishing, 3D refinement, and in the case of the collided di-ribosome, multi-body refinement.

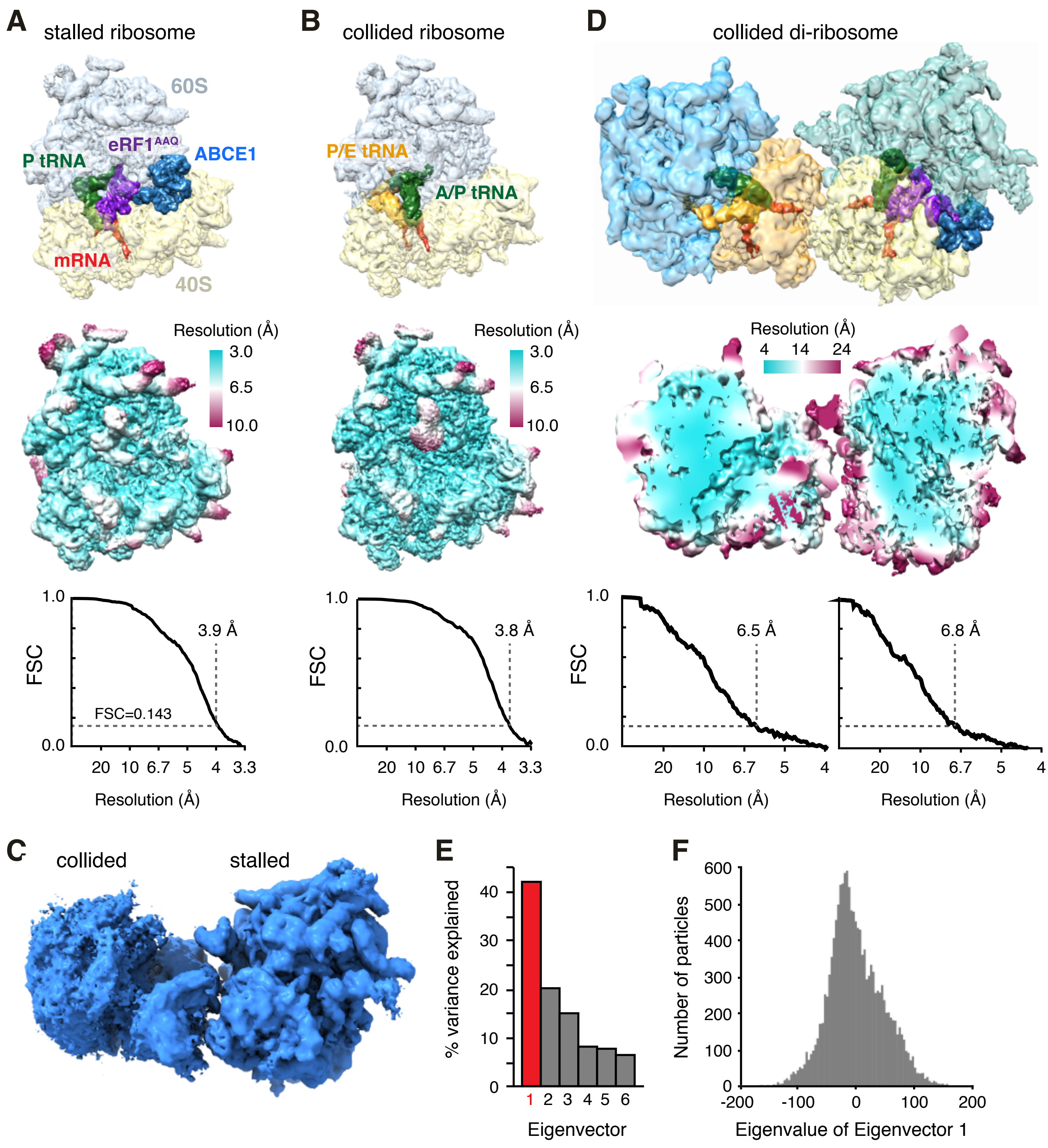

Fig. S4. Map Quality and Conformational Heterogeneity in Collided Di-ribosomes (related to Fig. 4). **(A, B)** Cryo-EM maps from this study (before post-processing) colored according to bound factors (top panels) and local resolution (middle panels). The bottom panels show Fourier shell correlation (FSC) curves computed between independent half-maps (black). The resolution estimated from map-to-map correlation at FSC = 0.143 is indicated. **(C)** Collided di-ribosome map with accurate alignment of the stalled ribosome. Notice how the rotation state, identity of bound factors and conformations of expansion segments of the collided ribosome are ambiguous due to structural heterogeneity. **(D)** The improved collided di-ribosome map resulting from multi-body refinement in which the stalled and collided ribosomes are treated independently. The middle panel shows local resolution through a section of the map, and the bottom panel shows FSC curves for the stalled and collided ribosomes in the map. **(E)** Histogram showing contributions of the top 6 eigenvectors from multi-body refinement and principal component analysis towards explaining structural variations among collided di-ribosome particles. **(F)** Histogram of number of particles versus eigenvalue for eigenvector 1 from panel E, which explains 42% of the variance in the structure of the collided di-ribosome.

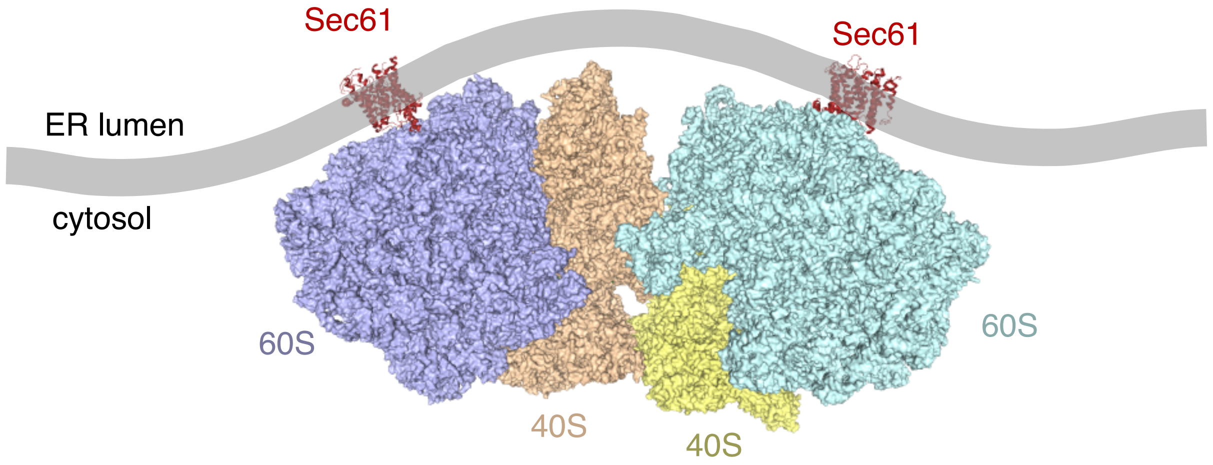

Fig. S5. Compatibility of the collided di-ribosome with membrane binding (related to Fig. 4).
A large proportion of total cellular translation occurs on ribosomes bound to the ER membrane. To determine whether the collided di-ribosome strucutre is compatible with membrane bound ribosomes, the Sec61 translocon (red) was docked into its binding site on each ribosome of the collided di-ribosome. The shaded bar depicts a hypothetical membrane bilayer, illustrating that it is compatible with the positions of Sec61 with some bending. This degree of membrane bending is easily within the range that is observed for ER membrane in electron micrographs. Furthermore, the flexibility of the di-ribosome at its interface (Fig. 4) may further facilitate collisions at the membrane surface.

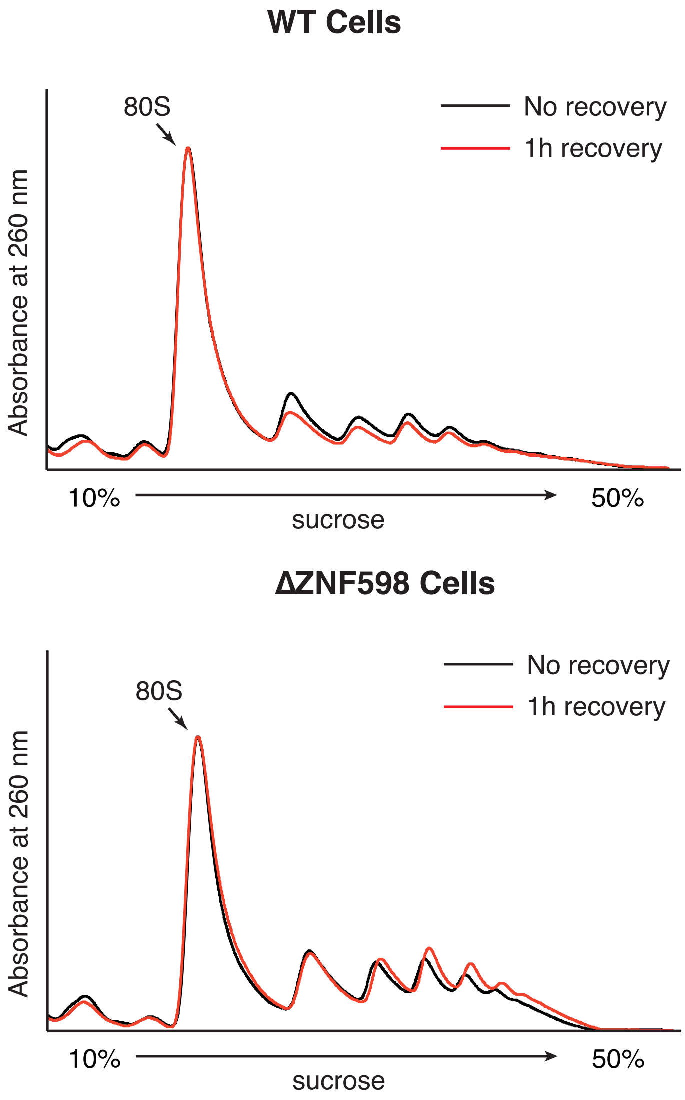

Fig. S6. Resolution of collided ribosomes relies on ZNF598 (related to Figure 5)
WT (top panel) or ΔZNF598 HEK293 cells were treated with 1.8 μM emetine for 15 minutes. One aliquot was harvested immediately, while another was allowed to recover for 1 hour in emetine-free medium before harvesting. Cytosol from each of the samples was digested with micrococcal nuclease and separated by sucrose gradient sedimentation. The absorbance at 260 nm across the gradient was plotted, normalized to the peak 80S absorbance. Note that the nuclease-resistant di-, tri-, and poly-ribosome peaks are partially resolved during the one hour recovery in WT cells, but persist in ΔZNF598 cells. In fact, the proportion of tetra-ribosomes and larger polysomes increases in ΔZNF598 cells, consistent with additional ribosome loading onto already stalled complexes.

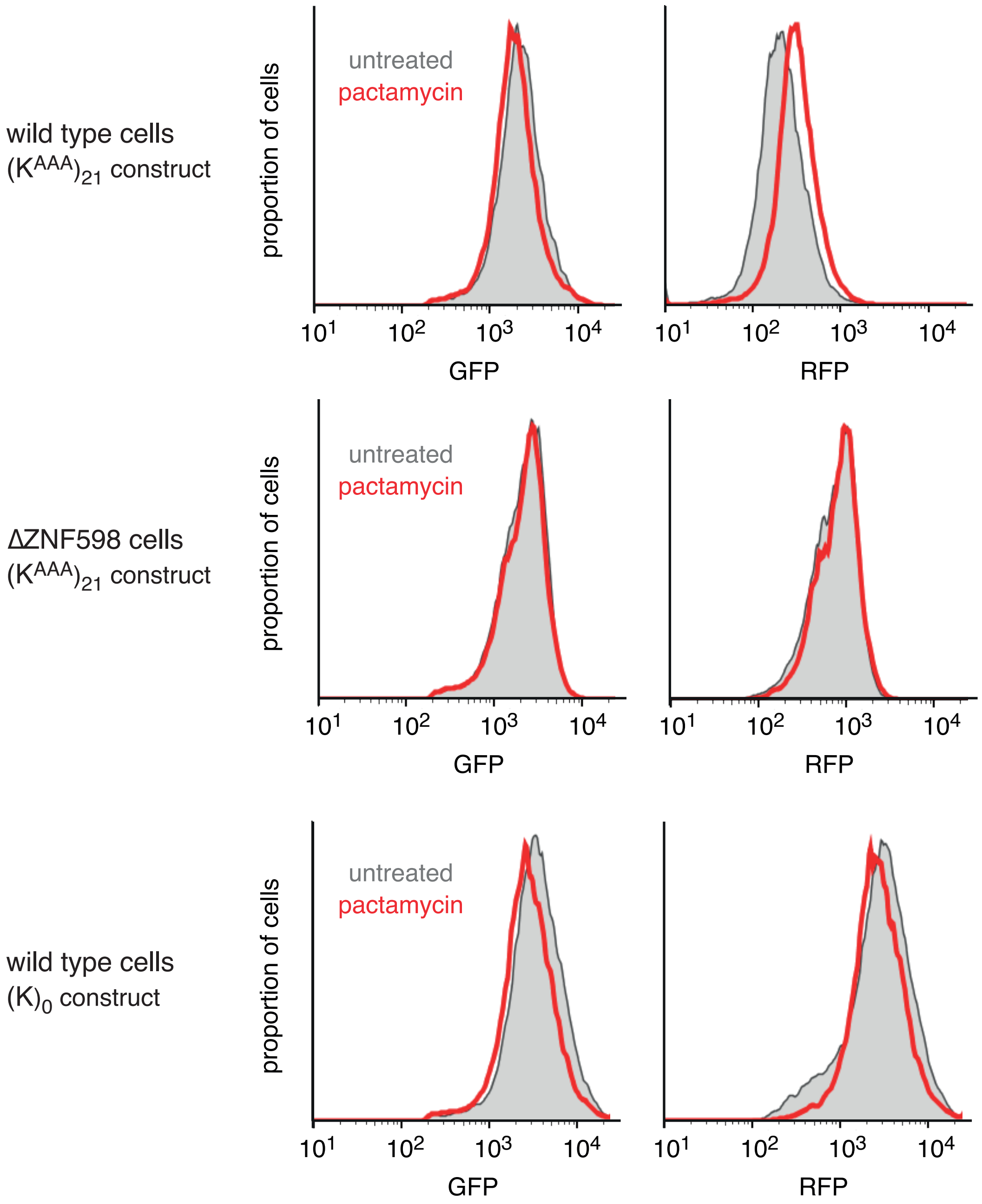

Fig. S7 Detection of ribosome stalling is context dependent (related to Figure 6)
Histograms for individual fluorescent protein signals from the indicated cells expressing the indicated constructs. The cells were either untreated or treated for 22 h with 10 nM pactamycin to partially inhibit initiation. Note that there is a selective increase in RFP expression only in wild type cells expressing $(K^{AAA})_{21}$ stalling reporter, which explains the observed increase in RFP:GFP ratio shown in Fig. 6B. Thus, the change in ratio can be ascribed to an increase in read-through of the poly(A) stalling sequence.

Table S1. Data collection, processing, refinement and model statistics (related to Figure 3).

	Stalled ribosome	Collided ribosome	Di-ribosome
**Data Collection**			
Voltage (kV)	300		-
Pixel size (Å)	1.07		-
Defocus range (µm)	-1.5 to -3.0		-
Defocus mean (µm)	-2.25		-
Electron dose (e^{-} $frame^{-1}$ $Å^{-2}$)	2.03, 1.55		
**Data Processing**			
Independent data collections	2		-
Useable micrographs	4815		-
Particle picking software	Gautomatch		-
Particles picked	364,776		-
Particles after 2D classification	257,331		-
Final particles	71,954	98,611	14,634
Map sharpening B-factor ($Å^2$)	-101.6	-86.3	-90.8(stalled)<br>-77.8(collided)
Resolution (Å)	3.9	3.8	6.8 (stalled)<br>6.5 (collided)
PDB accession code	6HCF	6HCJ	6HCM (stalled)<br>6HCQ (collided)
EMDB accession code	EMD-0192	EMD-0194	EMD-0195 (stalled)<br>EMD-0197 (collided)
**Refinement**			
Resolution (Å)	3.9	3.8	
Average B factor ($Å^2$)	75.4	96.7	
$FSC_{average}$	0.75	0.80	-
**R.M.S deviations**			
Bond lengths (Å)	0.019	0.013	-
Bond angles (°)	1.188	1.11	-
**Validation**			
Molprobity score	1.83	1.81	
Clashscore, all atoms (percentile)	5.27 (93rd)	4.53 (95th)	-
Favored rotamers (%)	94.9	95.0	-
Poor rotamers (%)	0.37	0.33	-
**Ramachandran plot**			
Favored (%)	90.0	88.9	-
Outliers (%)	0.2	0.34	-
**Validation (RNA)**			
Correct sugar puckers (%)	98.8	98.8	-
Good backbone conformations (%)	70	67	-
