# Supplementary material for: ZNF598 Is a Quality Control Sensor of Collided Ribosomes
Source: Mol Cell. 2018 Nov 1;72(3):469–481.e7. doi: 10.1016/j.molcel.2018.08.037 (PMC6224477; doi:10.1016/j.molcel.2018.08.037)
Supplement: Document S2. Article plus Supplemental Information [file mmc2.pdf]

Article

Molecular Cell

ZNF598 Is a Quality Control Sensor of Collided Ribosomes

Graphical Abstract

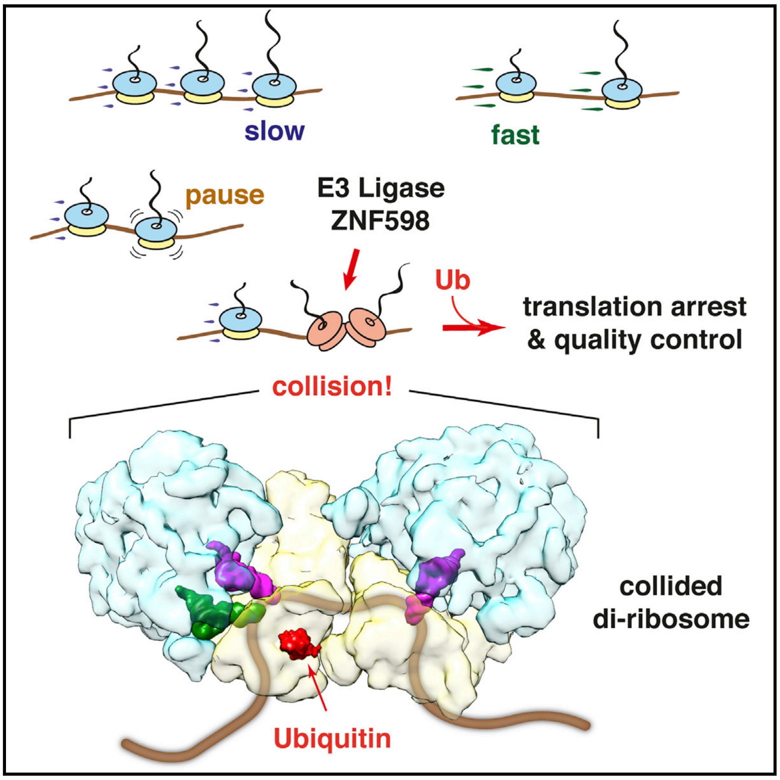

Highlights

- ZNF598 is a direct sensor of ribosome collisions incurred by many unrelated causes
- The minimal target recognized and ubiquitinated by ZNF598 is a collided di-ribosome
- Collided di-ribosome structure shows that ZNF598 ubiquitin sites are near the interface
- Collisions are required to terminally arrest translation in ZNF598-dependent manner

Authors

Szymon Juszkiewicz,
Viswanathan Chandrasekaran,
Zhewang Lin, Sebastian Kraatz,
V. Ramakrishnan, Ramanujan S. Hegde

Correspondence

vish@mrc-lmb.cam.ac.uk (V.C.),
rhegde@mrc-lmb.cam.ac.uk (R.S.H.)

In Brief

Cells contain millions of ribosomes that need to translate mRNAs accurately to maintain homeostasis. Ribosomes that slow excessively during translation must be promptly resolved to avoid disease. Juszkiewicz et al. show that ribosome collisions, a sign of aberrant translation, are detected by the ubiquitin ligase ZNF598 to initiate quality control.

Juszkiewicz et al., 2018, Molecular Cell *72*, 469–481
November 1, 2018 © 2018 MRC Laboratory of Molecular Biology.
Published by Elsevier Inc.
https://doi.org/10.1016/j.molcel.2018.08.037

CellPress

CellPress

ZNF598 Is a Quality Control Sensor of Collided Ribosomes

Szymon Juszkiewicz,[1,2] Viswanathan Chandrasekaran,[1,2,*] Zhewang Lin,[1] Sebastian Kraatz,[1] V. Ramakrishnan,[1] and Ramanujan S. Hegde[1,3,*]
[1]MRC Laboratory of Molecular Biology, Cambridge CB2 0QH, UK
[2]These authors contributed equally
[3]Lead Contact
*Correspondence: vish@mrc-lmb.cam.ac.uk (V.C.), rhegde@mrc-lmb.cam.ac.uk (R.S.H.)
https://doi.org/10.1016/j.molcel.2018.08.037

SUMMARY

Aberrantly slow translation elicits quality control pathways initiated by the ubiquitin ligase ZNF598. How ZNF598 discriminates physiologic from pathologic translation complexes and ubiquitinates stalled ribosomes selectively is unclear. Here, we find that the minimal unit engaged by ZNF598 is the collided di-ribosome, a molecular species that arises when a trailing ribosome encounters a slower leading ribosome. The collided di-ribosome structure reveals an extensive 40S-40S interface in which the ubiquitination targets of ZNF598 reside. The paucity of 60S interactions allows for different ribosome rotation states, explaining why ZNF598 recognition is indifferent to how the leading ribosome has stalled. The use of ribosome collisions as a proxy for stalling allows the degree of tolerable slowdown to be tuned by the initiation rate on that mRNA; hence, the threshold for triggering quality control is substrate specific. These findings illustrate how higher-order ribosome architecture can be exploited by cellular factors to monitor translation status.

INTRODUCTION

The ribosome has emerged as a major hub for both mRNA and protein quality control (Brandman and Hegde, 2016; Shoemaker and Green, 2012). Inability to complete the translation cycle successfully can be an indicator of many potential problems, including a damaged or improperly processed mRNA, insufficiency of a cognate acylated-tRNA, a faulty ribosome, or mutant translation factors. In each case, elongation slows or halts completely to activate pathways of ribosome recycling (Pisareva et al., 2011; Shoemaker et al., 2010), mRNA decay (Frischmeyer et al., 2002; van Hoof et al., 2002), nascent protein degradation (Ito-Harashima et al., 2007), stress responses (Brandman et al., 2012), and nutrient starvation (Marton et al., 1997). Prompt recognition and resolution of aberrant translation is crucial for maintaining cellular protein homeostasis (Choe et al., 2016; Izawa et al., 2017; Yonashiro et al., 2016) and avoiding disease (Chu et al., 2009; Ishimura et al., 2014). How aberrant translation is initially recognized by the cell to initiate downstream quality control and homeostatic responses is not well understood.

The challenge faced by the cell is to identify a relatively rare pathologic slowdown of translation amid widely heterogeneous normal translation speeds. Some types of translation stalls are straightforward to discriminate. For example, a ribosome that reads to the end of a truncated mRNA will contain an empty A site that cannot engage either elongation or termination complexes. Instead, ribosome rescue factors (Pelota-Hbs1 in mammals, Dom34-Hbs1 in yeast) bind effectively to an mRNA-lacking A site to initiate subunit separation (Becker et al., 2011; Pisareva et al., 2011; Shao et al., 2016; Shoemaker et al., 2010), which is required for subsequent degradation of mRNA (Tsuboi et al., 2012) and nascent chains (Shao et al., 2013). However, this type of stall is probably rare (Guydosh and Green, 2014), with most translation stalls occurring when the A site still contains mRNA.

The most frequent type of stall is probably the translation of poly(A). This scenario would arise when a nascent transcript is inappropriately polyadenylated within the coding region at a near-cognate poly(A) signal, resulting in a "non-stop" mRNA that lacks an in-frame stop codon (Frischmeyer et al., 2002; van Hoof et al., 2002; Kaida et al., 2010). Non-stop mRNAs are aberrant and are typically degraded by the cell (Frischmeyer et al., 2002; van Hoof et al., 2002). Recognition of a non-stop mRNA by quality control depends on its translation (Shoemaker and Green, 2012). It is thought that slowing of the ribosome during poly(A) translation (Koutmou et al., 2015; Lu and Deutsch, 2008) is a critical cue to initiating downstream mRNA and nascent protein degradation (Bengtson and Joazeiro, 2010; Dimitrova et al., 2009).

Recent experiments using reporters of poly(A)-mediated stalling in mammalian cells indicate a key role for site-specific ribosome ubiquitination in communicating aberrant translation to downstream quality control pathways (Garzia et al., 2017; Juszkiewicz and Hegde, 2017; Sundaramoorthy et al., 2017). The ubiquitin ligase ZNF598 and its target lysines on the 40S protein eS10 (and, to a lesser extent, uS10) are required to initiate quality control from poly(A) sequences. Hel2, the yeast homolog of ZNF598, and its target site on uS10 are similarly required to initiate quality control from poly-basic sequences and the difficult-to-decode CGA codons (Brandman et al., 2012; Letzring et al., 2013; Matsuo et al., 2017; Saito et al., 2015; Sitron et al.,

This is an open access article under the CC BY license (http://creativecommons.org/licenses/by/4.0/).

2017). Ubiquitination has been assumed to occur selectively on stalled ribosomes, but the basis of this presumed selectivity is unclear.

At least three models have been proposed for how cells might discriminate slow or stalled ribosomes from normal translation complexes. In the first model, a ribosome in the rotated state with hybrid-state tRNAs is the species that signals aberrant translation (Matsuo et al., 2017). This idea was supported by the stall site showing increased 22-nt ribosome-protected footprints (Matsuo et al., 2017) thought to reflect a rotated ribosome (Lareau et al., 2014). Furthermore, cryoelectron microscopy (cryo-EM) of ribosomes recovered with Hel2 showed enrichment for the rotated state (Matsuo et al., 2017). However, this model is difficult to reconcile with the fact that translating ribosomes spend roughly 25% of their time in a rotated state (Behrmann et al., 2015). This sub-population of normal ribosomes, which would exceed comparatively rare stalled ribosomes, should somehow need to avoid triggering quality control.

A second model posits that the decisive cue might be ribosomes containing multiple ^{Lys}(UUU) tRNAs and adjacent to a poly(A) sequence (Garzia et al., 2017). This idea was supported by the enrichment of both RNA species UV-crosslinked to ZNF598 in cultured cells. Although this model might explain poly(A)-mediated stalls, it would not be a general sensing mechanism that could explain other types of stalls known to depend on Hel2 for recognition. Furthermore, the observation of rRNA crosslinks that are spatially distant on the ribosome is difficult to rationalize with physical models of ZNF598 binding.

A third model proposed that ribosome collision behind a stalled ribosome is the decisive cue for triggering endonucleolytic cleavage of the stall-containing mRNA (Simms et al., 2017). This model was derived from the finding that several manipulations designed to reduce collisions led to reduced detection of the cleavage fragment. Whether the absence of a clear cleavage fragment under low-collision conditions reflected a failure of ribosomes to stall, a failure of cells to recognize the stall, or greater heterogeneity of cleavage sites was not investigated. Ribosome collisions were also correlated to Hel2-dependent ubiquitination of uS3 (Simms et al., 2017), but its relevance was ambiguous given that ribosome-associated quality control does not require uS3 ubiquitination (Matsuo et al., 2017). Because Hel2 seems to associate constitutively with both 80S monosomes and polysomes (Matsuo et al., 2017), its role in recognizing ribosome collisions versus acting at a later step has been unclear.

Despite these uncertainties, it is clear from genetic studies that ZNF598/Hel2 acts early during ribosome-associated quality control. The ability of ZNF598/Hel2 to modify and associate with ribosomes suggests it has a role in detecting translation stalling, but the molecular cues it might use for selective recognition have been a matter of speculation. We therefore investigated whether and how aberrantly slow translation through poly(A) sequences is selectively recognized by ZNF598. Our results show that ZNF598 specifically and directly detects collided ribosomes for site-specific 40S ubiquitination. The structure of the collided di-ribosome demonstrates how its recognition by ZNF598 can be universal to multiple types of stalls and suggests a general principle for how higher-order poly-ribosome architecture can convey translation status to quality control pathways.

RESULTS

ZNF598 Engages a Sub-population of Poly(A)-stalled Ribosomes *In Vitro*

To understand what ZNF598 might recognize as aberrant, we reconstituted its recruitment to poly(A)-stalled translation complexes in an *in vitro* system. Our preliminary characterization of the rabbit reticulocyte lysate (RRL) translation system showed extremely low levels of ZNF598 compared to cytosol from HEK293 cells (Figure S1A). A naturally ZNF598-deficient translation lysate provided a "blank slate" to which recombinant ZNF598 can be added to investigate its function. Thus, RRL was reconstituted with FLAG-tagged ZNF598 in the low nanomolar range, similar to the concentration in cultured HEK293 cells.

We translated an epitope-tagged poly(A)-containing transcript in the ZNF598-supplemented RRL system and affinity-purified translation complexes via the nascent protein (Figure 1A). Separation of the recovered complexes by sucrose gradient sedimentation revealed that ~70%–80% of ZNF598 co-fractionated with ribosomes (Figure 1B). The remainder was at the top of the gradient and had presumably dissociated during the size fractionation step that followed affinity enrichment. ZNF598, nascent chains, and ribosomes were not recovered from control reactions in which the nascent chains lacked the tag (data not shown).

The majority of poly(A)-stalled nascent chains were in the 80S monosome fractions, consistent with primarily a single round of initiation in the *in vitro* system. Very little ZNF598 was seen in these fractions, suggesting that it does not effectively engage these stalled 80S nascent chain complexes. This indicates that proximity to neither poly(A) nor ^{Lys}(UUU) tRNAs in the ribosome are sufficient for ZNF598 recognition as speculated previously (Garzia et al., 2017). Instead, ZNF598 co-fractionated primarily with a minor population of nascent chains that migrated deeper into the gradient, corresponding to di-ribosomes. Minimizing multiple rounds of translation with the initiation inhibitor pactamycin reduced both the di-ribosome population and ZNF598 recruitment to ribosomes (Figure S1C). Thus, ZNF598 engages di-ribosome complexes more effectively than stalled 80S ribosomes.

Affinity purification of a poly(A)-stalled translation reaction via ZNF598 led to the same conclusion. ZNF598 co-precipitated stalled nascent chains of two predominant sizes (Figure 1C). The shorter nascent chains were a relatively minor species in the total translation reaction but reached comparable abundance to the primary stalled nascent chain population in the ZNF598-purified sample. Fractionation of the input and affinity-purified samples by sucrose gradient sedimentation showed that the shorter nascent chains migrate in the di-ribosome fractions. Thus, these nascent chains apparently represent the products of ribosomes queued behind the primary stalled ribosome. This di-ribosome population was selectively recovered with ZNF598, explaining the strong enrichment of shorter nascent chain products. Quantification showed that the nascent chain products recovered via ZNF598 had the same migration profile as di-ribosome-specific truncated nascent chains. These results indicate that a single ribosome stalled on poly(A) is insufficient for stable recruitment of ZNF598; instead, ZNF598 preferentially engages the minor population of di-ribosome translation complexes.

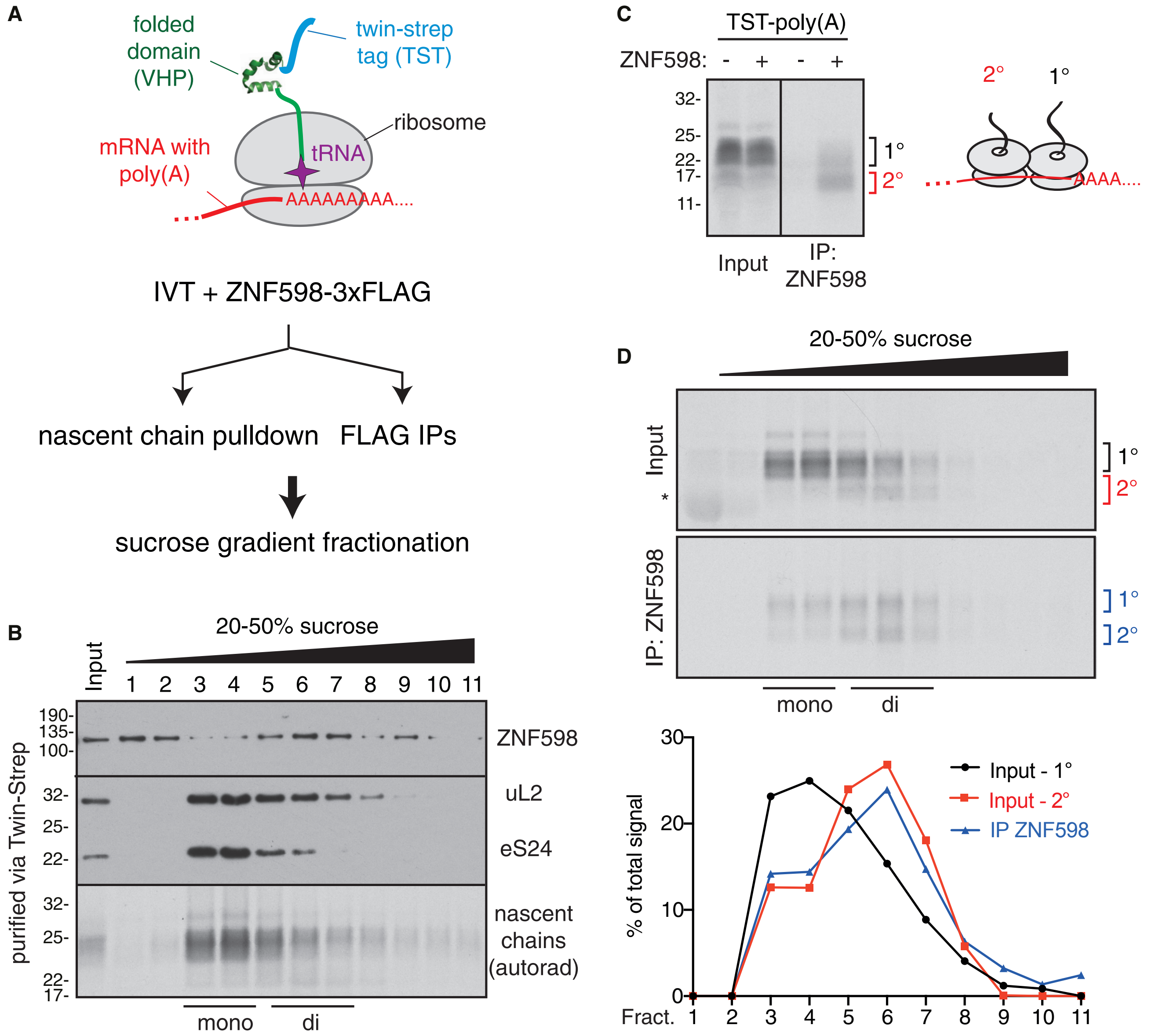

Figure 1. ZNF598 Engages a Sub-population of Poly(A)-Stalled Ribosomes *In Vitro*

(A) Strategy to analyze poly(A)-stalled ribosome-nascent chain complexes produced by *in vitro* translation (IVT). VHP is a small autonomously folding three-helix bundle from the villin head piece.

(B) Poly(A)-stalled translation complexes were affinity purified via the nascent chain and separated by sucrose gradient sedimentation. The affinity-purified products (input) and each gradient fraction were analyzed by autoradiography (to detect nascent chains) or immunoblotting for recombinant ZNF598 or ribosomal proteins uL2 and eS24. Mono- and di-ribosome fractions are indicated.

(C) Poly(A)-stalled translation complexes from reactions lacking or containing 5 nM FLAG-tagged ZNF598 were immunopurified via the FLAG tag, and the nascent chains were detected by autoradiography. 1° and 2° indicate the position of nascent chains from the first and second ribosome of the stalled complexes (see diagram).

(D) The input and affinity-purified samples prepared as in (C) were separated by sucrose gradient and the nascent chains detected by autoradiography. The graph below the autoradiograms depicts the distribution of nascent chains corresponding to mono-ribosomes (1°, black) or di-ribosomes (2°, red) in the input sample or the nascent chains recovered by affinity purification ("IP ZNF598" - blue).

See also Figure S1.

Site-Specific Stalling of Native Polysomes Triggers ZNF598 Engagement

The low yield of higher-order ribosome complexes on exogenous transcripts limited the utility of this system for biochemical and structural studies. We therefore developed a strategy to cause site-specific translation stalling on the abundant globin-translating native poly-ribosomes in RRL. We exploited eRF1^{AAQ}, a dominant-negative mutant release factor (Brown et al., 2015a;

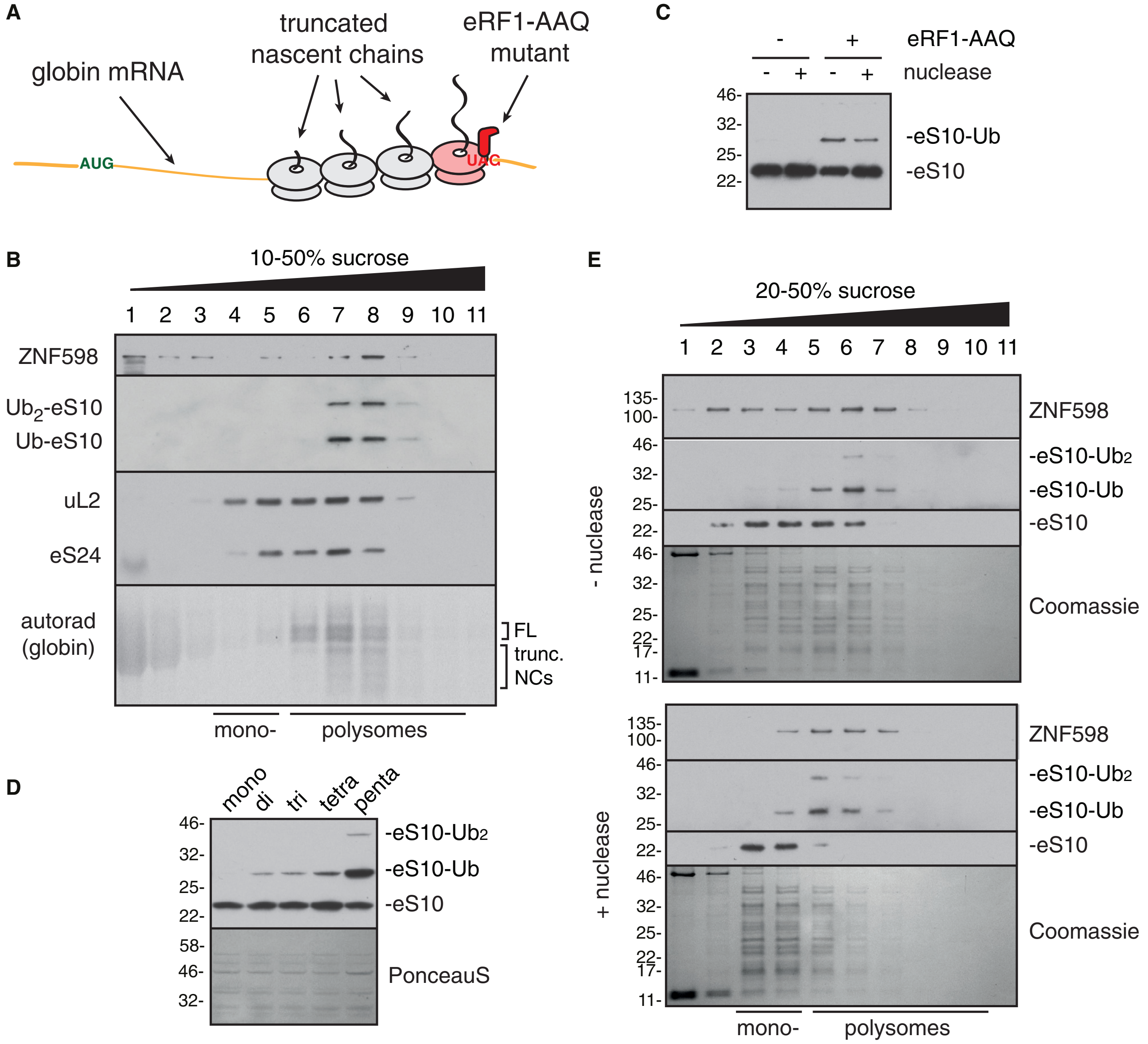

Figure 2. Site-Specific Stalling of Native Polysomes Triggers ZNF598 Engagement

(A) Strategy to site-specifically stall ribosomes at the stop codon on native polysomes translating globin mRNA using the mutant release factor eRF1^{AAQ}.

(B) The polysomes in native reticulocyte lysate (RRL) were allowed to elongate in a translation reaction supplemented with 1 μM eRF1^{AAQ}, 5 nM FLAG-tagged ZNF598, and 10 μM His-tagged ubiquitin. After fractionation on a sucrose gradient, translated globin polypeptides were detected by autoradiography and the other products by immunoblotting. Ubiquitinated eS10 was detected with anti-eS10 after pull-down via the His-tag on ubiquitin. FL indicates full-length nascent chains; "trunc. NCs" are truncated nascent chains.

(C) Native RRL polysomes elongated in the absence or presence of 1 μM eRF1^{AAQ} were incubated without or with micrococcal nuclease, ubiquitinated with ZNF598, and analyzed for eS10 by immunoblotting.

(D) Native RRL polysomes stalled with eRF1^{AAQ} were fractionated by a high resolution sucrose gradient (see Figure S2B). Fractions enriched in mono-, di-, tri-, tetra-, and penta-ribosomes were normalized to contain an equal number of ribosomes, ubiquitinated with 10 nM ZNF598, and analyzed by immunoblotting for eS10. The stained blot verifies equal input ribosomes in each reaction.

(E) Reactions corresponding to the third and fourth lanes of (C) were fractionated on a sucrose gradient and analyzed by Coomassie staining or immunoblotting. The positions of mono- and poly-ribosomes are indicated. The primary bands seen in the stained gel are ribosomal proteins.

See also Figure S2.

Shao et al., 2016), to induce a local translation arrest at the stop codon (Figure 2A). The primary nascent chains produced in the eRF1^{AAQ}-containing translation reaction comprised full-length globin (Figure 2B). Approximately half of these nascent chains co-sedimented with ribosomes while the remainder of full-length products migrated in ribosome-free fractions and represent

polypeptides terminated by endogenous eRF1. The ribosome-associated nascent chains migrated mostly in the di-, tri-, and larger poly-ribosome fractions, consistent with the presence of multiple ribosomes on these mRNAs. The lower molecular weight nascent chains in these fractions (Figure 2B) are the products of ribosomes queued 5′ of the trapped termination complex and were not seen when eRF1^{AAQ} was omitted (Figure S2A).

ZNF598 that was included in the eRF1^{AAQ}-stalled reaction co-migrated predominantly with polysomes, in which ubiquitinated eS10 was observed. Some apparent dissociation during sucrose gradient separation probably accounts for the small amount of ZNF598 seen in the lower molecular weight fractions. Importantly, ZNF598 was detected primarily in the non-ribosomal fractions when translation was allowed to run off (Figure S2A). Thus, localized stalling of endogenous polysomes at the termination codon by eRF1^{AAQ} produces higher-order ribosome complexes that are targets for ZNF598 engagement and eS10 ubiquitination.

A Nuclease-Resistant Di-ribosome Is the Minimal ZNF598 Target

Post-translational addition of ZNF598 after eRF1^{AAQ}-mediated stalling led to eS10 ubiquitination (Figure 2C) similar to the reaction when ZNF598 was included co-translationally (Figure 2B). Importantly, eS10 ubiquitination was not observed in the absence of eRF1^{AAQ}, demonstrating that stalling was needed for engagement by ZNF598. Ubiquitination reactions of ribosome complexes containing from one to five ribosomes (Figure S2B) showed that di- to penta-ribosome complexes were ubiquitinated on eS10 while monosomes were not (Figure 2D). Of note, the proportion of eS10 that was ubiquitinated increased for complexes containing more ribosomes. For example, penta-ribosomes were more effectively ubiquitinated on a per-ribosome basis than di-ribosomes. This observation is consistent with a model in which each trailing ribosome in the queue can be ubiquitinated by ZNF598.

To further establish that higher-order ribosome complexes are the preferred target for ZNF598, we digested the stalled complexes with nuclease to disassemble polysomes before addition of ZNF598. Unexpectedly, ubiquitination was essentially unaffected by nuclease pre-treatment (Figure 2C). Sucrose gradient fractionation verified that nearly all polysomes had been effectively converted to monosomes by nuclease digestion (Figures 2E and S2C). However, a minor population of polysomes proved resistant to nuclease treatment, and this population preferentially engaged ZNF598 (as judged by co-sedimentation) and was ubiquitinated on eS10. Remarkably, almost all of the eS10 in this minor nuclease-resistant polysome fraction was modified, while essentially no modification was observed in the monosome population (Figure S2C). This result suggested that tightly packed (and hence, nuclease-resistant) poly-ribosomes, but not individual stalled ribosomes or polysomes that are loosely packed, are the preferred target for ZNF598 ubiquitination.

The Consensus Structure of a Native Stalled Di-ribosome

The tetra-ribosome peak of queued ribosomes stalled with eRF1^{AAQ} (but lacking ZNF598) was analyzed by single-particle cryo-EM. Although this specimen did not contain monosomes when analyzed by repeat sucrose gradient fractionation, the EM images showed isolated ribosomes in addition to polysomes (Figure S3A). This suggested that polysomes disassembled to some degree during vitrification, necessitating an *in silico* classification strategy to identify intact collided ribosome complexes (Figure S3B). Using focused classification with partial signal subtraction, we identified the subset of ribosomes that contained eRF1^{AAQ} and ABCE1. From this subset of “stalled” ribosomes, we identified the population that contained an adjacent collided ribosome, re-extracted and re-centered these particles, and used them to construct an initial map of a collided di-ribosome.

Although the map reveals a defined configuration of the two ribosomes, flexibility at the inter-ribosomal interface was evident: masking the stalled ribosome worsened the resolution of the collided ribosome (e.g., Figure S4C) and vice versa (data not shown). We therefore used multi-body refinement (Nakane et al., 2018) to treat the stalled and collided ribosomes as separate rigid bodies. This approach produced an improved collided di-ribosome map with the stalled and collided ribosomes at 6.8 Å and 6.5 Å resolution, respectively (Figure S4D).

Using all the ribosome particles from this same dataset as a starting point, we generated higher resolution maps from the two major classes of particles: those containing eRF1^{AAQ} and ABCE1 (at 3.9 Å resolution) and those in a rotated state with hybrid tRNAs (at 3.8 Å resolution). These two maps proved to be higher-resolution versions of the stalled and collided ribosomes in the collided di-ribosome map, consistent with their origin from a collided complex that disassembled partially during vitrification. Thus, the higher-resolution versions were used to build atomic models that were then docked into the appropriate positions of the collided di-ribosome map (Figure 3A).

The stalled ribosome is observed in the canonical state with peptidyl-tRNA in the P site. As expected, accommodated eRF1^{AAQ} is observed in the A site, and ABCE1 is observed at the factor-binding site (Figure 3A). Unlike the earlier 80S•eRF1^{AAQ}•ABCE1 monosome structure (Brown et al., 2015a), the same complex in the collided di-ribosome lacks E-site tRNA and displays an “open” conformation of the L1 stalk. The adjacent collided ribosome is observed in the rotated state with A/P and P/E hybrid tRNAs and an empty factor-binding site. Although this conformation should be competent for eEF2 binding, no density was observed even after focused classification with signal subtraction, suggesting that the adjacent stalled ribosome may sterically hinder eEF2 accessibility. The inter-ribosomal interface is formed by small subunit interactions involving both the head and the body (Figure 3A). Two regions of close juxtaposition between the 40S subunits are observed, which we refer to as interface 1 (Figure 3B) and interface 2 (Figure 3C). Both interfaces are at noteworthy regions and help explain earlier biochemical, genetic, and functional data on ribosome stalling.

Interface 1 involves eS1, uS11, eS26, and eS28 of the stalled ribosome. These four proteins are observed surrounding the mRNA at the mouth of the mRNA channel exit (Figure 3B). They contact elements near the mRNA channel entrance on the collided ribosome. Helix 16 from the18S rRNA of the collided ribosome contacts uS11, eS26, and eS28, while uS4 from the collided ribosome contacts eS1. In addition, uS3, another protein at the collided ribosome's mRNA channel entrance, is part

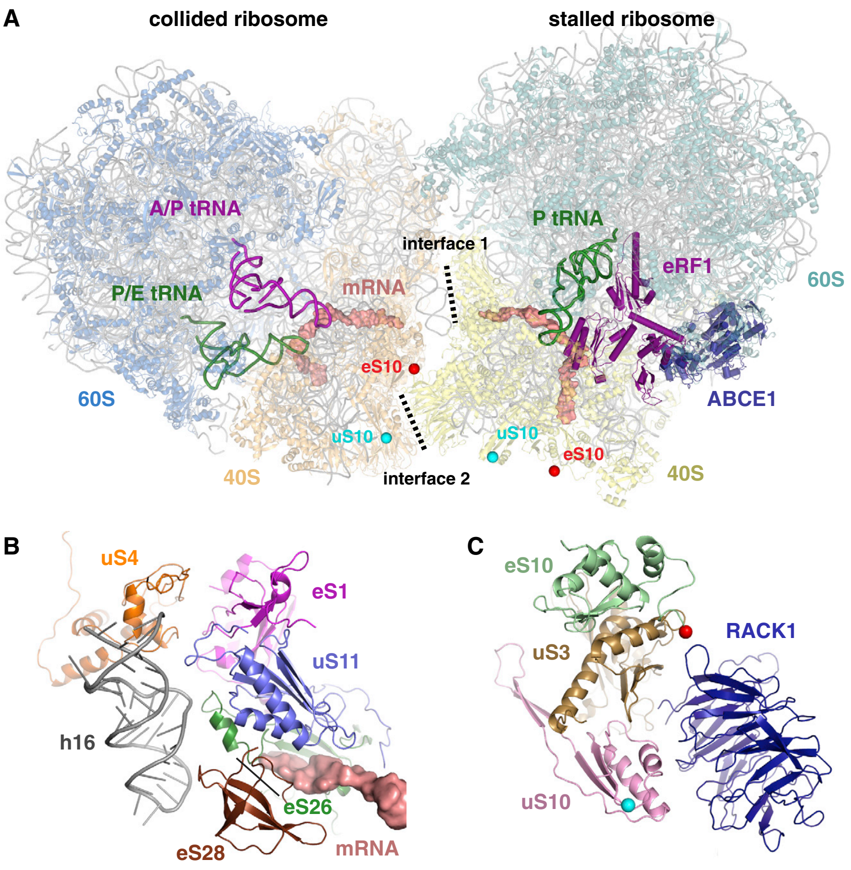

Figure 3. Structure and Interfaces of the Collided Di-ribosome

(A) Overview of the collided di-ribosome structure. The two primary interfaces between the 40S subunits of the stalled and collided ribosomes are indicated. Red and cyan balls indicate the approximate sites of ubiquitination on eS10 and uS10, respectively. The ubiquitinated residues are not modeled, so the most proximal visible residues are indicated.

(B) Close-up view of interface 1. Proteins eS1, uS11, eS26, and eS28 are from the stalled ribosome and make contact with uS4 and h16 of the 18S rRNA of the collided ribosome. The mRNA in the stalled ribosome channel is shown.

(C) Close-up of interface 2 showing the approximate sites of eS10 and uS10 ubiquitination as in (A). RACK1 is from the stalled ribosome, while uS3, eS10, and uS10 are from the collided ribosome.

See also Figures S3 and S4 and Table S1.

of interface 2 (Figure 3C) as discussed below. Thus, not only are the mRNA channels of the stalled and collided ribosomes closely juxtaposed, but this region is also stabilized by interactions across the inter-ribosomal interface. This architecture explains the observed nuclease resistance of collided di-ribosomes in biochemical assays. Furthermore, the 9 codon distance between the two ribosomal P sites imposed by the di-ribosome structure, together with translocation in 3-nt steps, may trap the collided ribosome in a rotated state. This feature, together with partial polysome dissociation during vitrification, could explain the earlier observation that Hel2 affinity purification from yeast lysate was enriched for rotated ribosomes (Matsuo et al., 2017).

Interface 2 is formed by RACK1 of the stalled ribosome and uS3, uS10, and eS10 of the collided ribosome. All four of these proteins have been biochemically or functionally implicated in ribosome stalling and quality control. RACK1 is an integral part of interface 2, and it is likely that the stability of a collided di-ribosome would be compromised in its absence. This consequence could explain why RACK1 depletion impairs detection of ribosome stalling in both yeast and mammals (Brandman et al., 2012; Matsuo et al., 2017; Sitron et al., 2017; Sundaramoorthy et al., 2017).

In mammals, the flexible C-terminal tail of eS10 is the primary target for ZNF598 ubiquitination (Garzia et al., 2017; Juszkiewicz and Hegde, 2017; Sundaramoorthy et al., 2017), while the N-terminal tail of uS10 is the primary target for Hel2 in yeast (Matsuo et al., 2017). Although the residues that get ubiquitinated are not visible in our structure, the closest visible residues from both proteins in the collided ribosome are near interface 2 (Figure 3C). By contrast, the same sites on the stalled ribosome are >50 Å from the interface (Figure 3A).

Ubiquitination of lysine 212 of uS3 appears to be dependent on uS10 ubiquitination by Hel2 (Matsuo et al., 2017). While the role of uS3 in quality control is not clear, its ubiquitination is affected by organelle and translation stress (Higgins et al., 2015). Lysine 212 of uS3 from the stalled ribosome is located at the interface. Thus, all the 40S ubiquitination sites implicated directly or indirectly in quality control are at interface 2. The selectivity of ZNF598 for multi-ribosome complexes over stalled monosomes argues that the interface region is recognized. We favor a model in which ubiquitination occurs on eS10 or uS10 of the collided ribosome and, perhaps in second step, uS3 of the stalled ribosome.

Tolerance and Flexibility of the Di-ribosome Interface

Although the eRF1^{AAQ}-stalled ribosome is in the canonical state, other impediments to elongation may stall ribosomes in a rotated state. A general system for detecting stalling would thus need to recognize both situations. When we computationally replaced the stalled ribosome in the di-ribosome structure with a rotated state ribosome, no obvious clashes are observed between the 60S subunits (Figure 4A). The 40S subunit changes very little (RMSD of only 1.4 Å), thereby maintaining a similar 40S-40S interface that could be effectively recognized by ZNF598. In support of this conclusion, a stalled tri-ribosome appears to be ubiquitinated more effectively than the stalled di-ribosome (Figure 2E). Given that the second ribosome of the tri-ribosome is rotated (Figure 3A), its interface with the third must necessarily

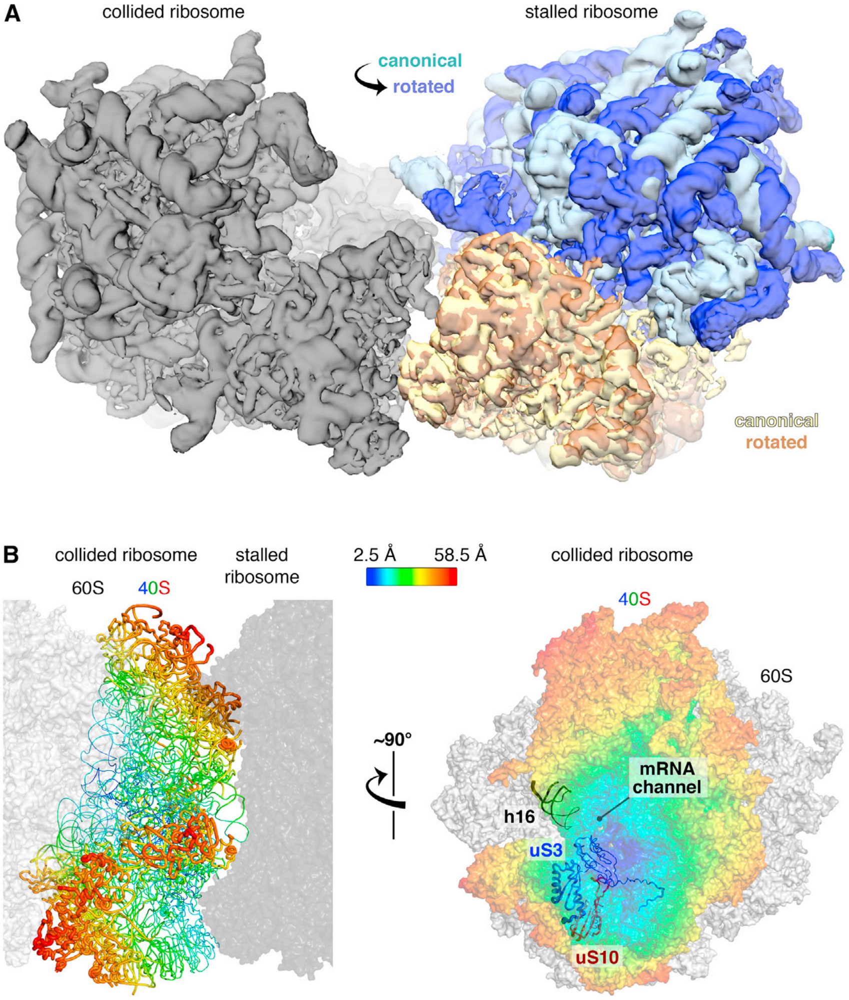

Figure 4. Tolerance and Flexibility of the Collided Di-ribosome

(A) Maps of canonical-state (pale cyan/yellow) and rotated-state (blue/orange) ribosomes are superimposed in the "stalled" position of the collided di-ribosome structure using the 40S subunits for alignment. The 40S interfaces are relatively invariant to rotation state and the 60S subunits do not clash with the collided ribosome (gray).

(B) Multi-body refinement and principal component analysis (Figures S4E and S4F) were used to quantify the nature and extent of heterogeneity in the relative positions of the stalled and collided ribosomes in di-ribosome particles. Maximal variance of the 40S subunit of the collided ribosome relative to a fixed stalled ribosome (dark gray) is displayed as a heatmap in sausage (left) or surface (right) representation. The interfaces and areas around the mRNA channel show the least variance.

See also Figures S4 and S5.

be different than its interface with the first, canonical-state ribosome. The observation that this two-interface complex is ubiquitinated more effectively than the di-ribosome with only one interface suggests that both types of interface are recognized by ZNF598.

The consensus di-ribosome structure represents an average configuration of an ensemble of conformations. Principal-component analysis was used to identify and rank the major movement modes that best explained the variance in orientations (Figures S4E and S4F). The largest contribution (42%) to the variance corresponded to a pivoting of the two monosomes about an axis roughly parallel to the length of the 40S subunits (Figure 4B). The mRNA connecting the two ribosomes remains buried throughout the range of movement, and the vicinity of eS10 and uS10 moves the least. Thus, the interface in which ZNF598 is likely to act has a limited range of motion. This di-ribosome flexibility probably explains why mRNA density between the two ribosomes is not visible in our structure. Biologically, the flexibility may facilitate accommodation of different translation states of the stalled ribosome, permit longer helical queues of ribosomes (Myasnikov et al., 2014), or help tolerate membrane-bound ribosome collisions (Figure S5).

Detection of ZNF598-Dependent Collided Di-ribosomes *In Vivo*

Based on the biochemical and structural analyses, the collision event that correlates with ZNF598 recognition is not simply proximity, but a defined conformation that fully shields the inter-ribosomal mRNA. We used this property to determine whether the same conformation is likely to be generated *in vivo* by ribosome collision. To induce collisions in cells, we used sub-inhibitory concentrations of emetine, an elongation inhibitor that is effectively irreversible (Grollman, 1968). These conditions should stochastically inhibit a subset of ribosomes and allow non-inhibited ribosomes to catch up to inhibited ones (Figure 5A). At fully inhibitory concentrations, emetine would stall all ribosomes before collisions could occur.

As expected, the distribution of polysomes in untreated or fully inhibited cells collapsed almost entirely to monosomes upon digestion with micrococcal nuclease (Figure 5B). By contrast, inhibition at low emetine concentrations resulted in nuclease-resistant populations of di-, tri-, and other higher-order ribosomal complexes, consistent with the tight packing seen in the collided di-ribosome structure. These nuclease-resistant polysomes could be partially resolved in a ZNF598-dependent manner (Figure S6) and accumulated to higher levels in cells lacking ZNF598 (Figure 5B). Thus, nuclease-resistant collided ribosomes are resolved by a ZNF598-dependent pathway *in vivo*.

ZNF598 Detects Collided Ribosomes Induced by Multiple Types of Stalls *In Vivo*

A major implication of the *in vitro* and structural analysis of stalled translation complexes is that recognition by ZNF598 should be impervious to the basis of stalling and configuration of the stalled ribosome (Figure 4A). To test this model in cells, we used sub-inhibitory concentrations of different translation inhibitors that

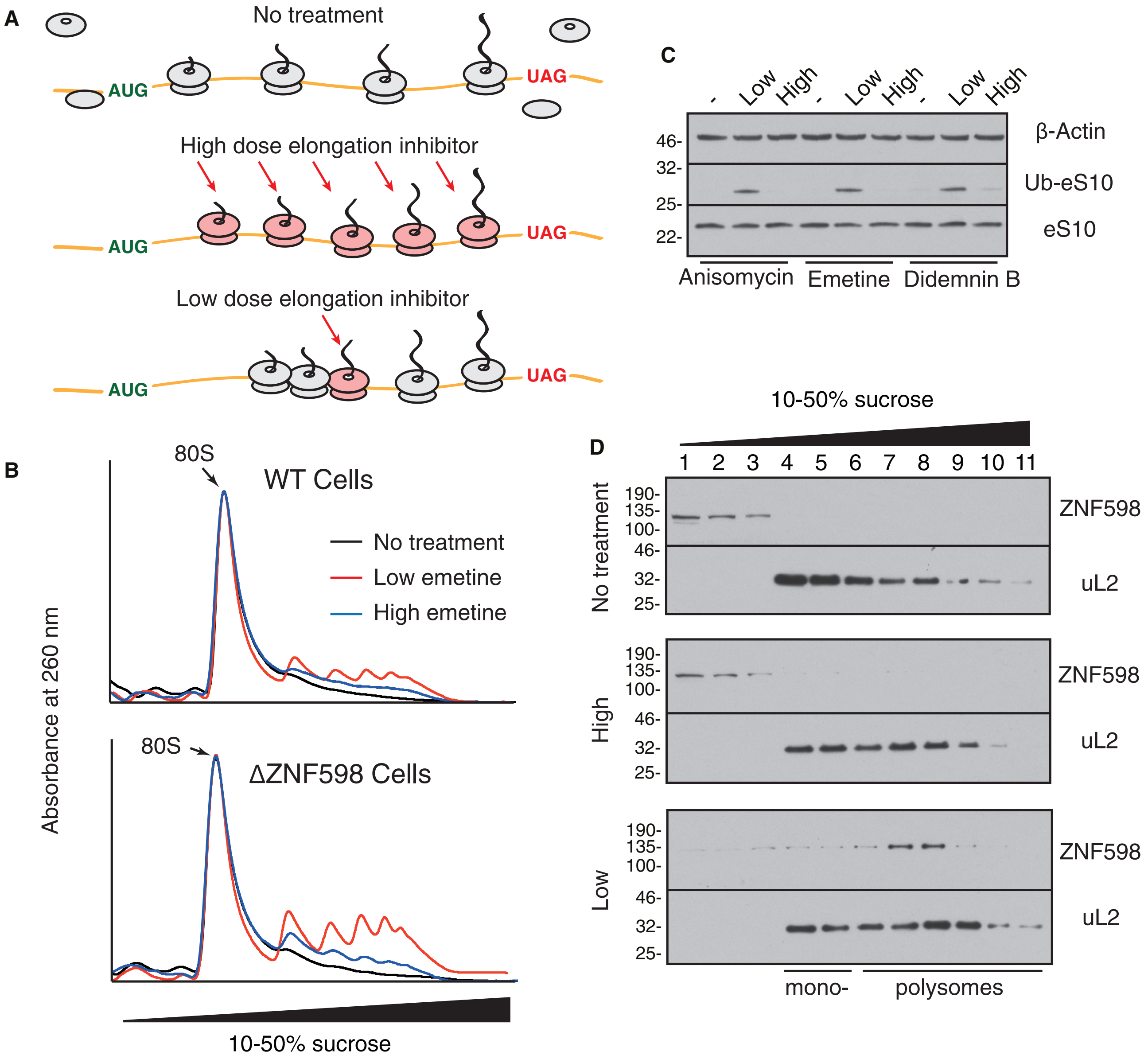

Figure 5. ZNF598 Detects Collided Ribosomes Induced by Multiple Types of Stalls *In Vivo*

(A) Strategy to induce complete versus stochastic ribosome stalling in cells treated with translation elongation inhibitors.

(B) Wild-type (WT) or ZNF598-knockout (ΔZNF598) HEK293 cells were pre-treated for 15 min with low (1.8 μM) or high (360 μM) emetine, lysed, digested with micrococcal nuclease, and separated by sucrose gradient centrifugation. The 260 nm absorbance profiles across the gradient are normalized to the 80S mono-ribosome peak.

(C) HEK293 cells pre-treated for 15 min with low or high concentrations of the indicated elongation inhibitors were analyzed by immunoblotting. Ubiquitinated eS10 (Ub-eS10) was detected using more sensitive reagents than unmodified eS10. For reference, ~10% of total eS10 is ubiquitinated in low-dose inhibitor-treated samples and ~1%–2% in untreated samples.

(D) Cytosol from HEK293 cells pre-treated for 15 min with nothing (top), high (middle), or low (bottom) concentrations of emetine were separated by sucrose gradient fractionation and immunoblotted for ZNF598 and uL2.

See also Figure S6.

stall ribosomes at different steps of elongation. In untreated HEK293 cells at steady state, eS10 ubiquitination levels are very low (Figure 5C) and little or no endogenous ZNF598 is ribosome associated (Figure 5D). Acute stalling of all ribosomes with fully inhibitory concentrations of three different elongation inhibitors (anisomycin, emetine, and didemnin B) resulted in very little increase in eS10 ubiquitination. By contrast, eS10 ubiquitination was markedly elevated at sub-inhibitory concentrations (Figure 5C). As shown for one of the inhibitors, ZNF598 recruitment to ribosomes mirrored the ubiquitination status of eS10 (Figure 5D).

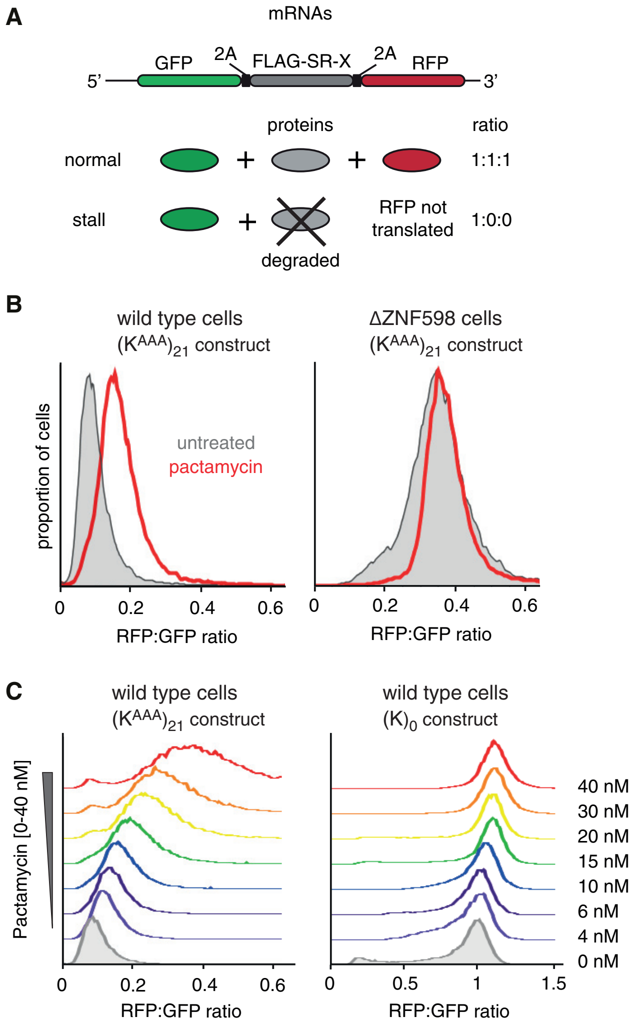

Figure 6. Detection of Ribosome Stalling Is Context Dependent

(A) Reporter construct and expected protein products in the absence or presence of terminal stalling. FLAG-SR-X is a "stalling reporter" in which X represents either a stalling sequence (poly(A), coding for 21 Lysine codons, termed $(K^{AAA})_{21}$) or no insert (termed $(K)_0$). The RFP:GFP ratio is a quantitative measure of terminal stalling.

(B) Histograms of the RFP:GFP ratios quantified by flow cytometry of WT or ΔZNF598 HEK293 cells expressing the $(K^{AAA})_{21}$ containing reporter. The cells were induced to express the reporter for 22 hr in the presence (red traces) or absence (gray traces) of 10 nM pactamycin, an inhibitor of translation initiation.

(C) HEK293 cells expressing the $(K^{AAA})_{21}$ stalling reporter (left) or control $(K)_0$ construct (right) were cultured for 22 hr in the absence (shaded gray trace) or presence of varying concentrations of pactamycin (colored traces). Staggered histograms of the RFP:GFP ratio measured by flow cytometry are plotted.

See also Figure S7.

Most of the ribosome-associated ZNF598 was observed in polysome fractions, consistent with the ZNF598 interaction with higher order ribosome complexes *in vitro*.

Anisomycin inhibits peptidyl transfer (Barbacid et al., 1975), emetine inhibits translocation (Jiménez et al., 1977), and didemnin B inhibits eEF1 dissociation from the ribosome (Shao et al., 2016). Thus, the ribosome would be stalled in three different states with respect to intersubunit rotation, position of tRNAs, and occupancy of the A site and GTPase center. Yet, none of these acutely stalled ribosomes is able to recruit ZNF598 for eS10 ubiquitination, supporting our conclusion that the stall itself is not recognized. Instead, a shared consequence of each type of stall appears to be recognized. Given our biochemical results and the observation that ZNF598 recruitment and eS10 ubiquitination require incomplete translation inhibition, this shared consequence is ribosome collisions.

Detection of Ribosome Stalling Is Context Dependent

A major implication of collisions being used to detect stalling is that translation dynamics will influence whether or not a given stall is recognized as aberrant by the cell (Simms et al., 2017). For example, an mRNA that is translated frequently will have an average inter-ribosomal distance that is shorter than an mRNA that is translated infrequently. Thus, the extent of slowdown that is tolerated by these two messages before a ribosome collision occurs will be different. Conversely, it should be possible to allow increased read-through of a transient stall simply by reducing the frequency of initiation.

To test this prediction, we analyzed a dual-fluorescence translation reporter containing a poly(A) stalling sequence (Juszkiewicz and Hegde, 2017). Under normal conditions, the upstream fluorescent protein (GFP) is translated, but stalling at the poly(A) sequence and ZNF598-dependent engagement of quality control precludes translation of the downstream fluorescent protein (RFP) (Figure 6A). Thus, the RFP:GFP ratio is markedly lower than for a matched reporter lacking the poly(A) stretch. Partial inhibition of translation initiation with pactamycin resulted in a clear increase in poly(A) read-through as judged by an increase in the RFP:GFP ratio (Figure 6B) attributable to an increase in RFP (Figure S7). Importantly, this effect was not seen in cells lacking ZNF598 (Figure 6B). Titration of pactamycin caused a dose-dependent increase in read-through of poly(A) but had a minimal effect on the non-stalling control (Figure 6C). Thus, the efficacy with which ZNF598-mediated quality control is induced from a poly(A) stall is influenced by the frequency of initiation, consistent with the collision-sensing model for ZNF598 function.

DISCUSSION

This study shows that the ubiquitin ligase ZNF598 is a direct sensor of ribosome collisions that occurs when a trailing ribosome "catches up" to an aberrantly slow leading ribosome. The collided di-ribosome recognized by ZNF598 is not simply two ribosomes tethered on a shared mRNA, but a well-defined structural unit. The higher-order complex formed by two juxtaposed ribosomes generates a distinctive but sufficiently flexible 40S-40S interface compatible with different translation states of the stalled ribosome. Thus, many types of aberrant translation can be detected by the same unifying mechanism.

The two primary targets of ZNF598 (eS10 and uS10) on the 40S subunit are observed at the di-ribosome interface. Considering that the interface is the least mobile and most distinctive feature of the di-ribosome, it is very likely to contain the ZNF598-binding site. A collided di-ribosome structure containing ZNF598 will be needed to define its interaction in molecular

CellPress

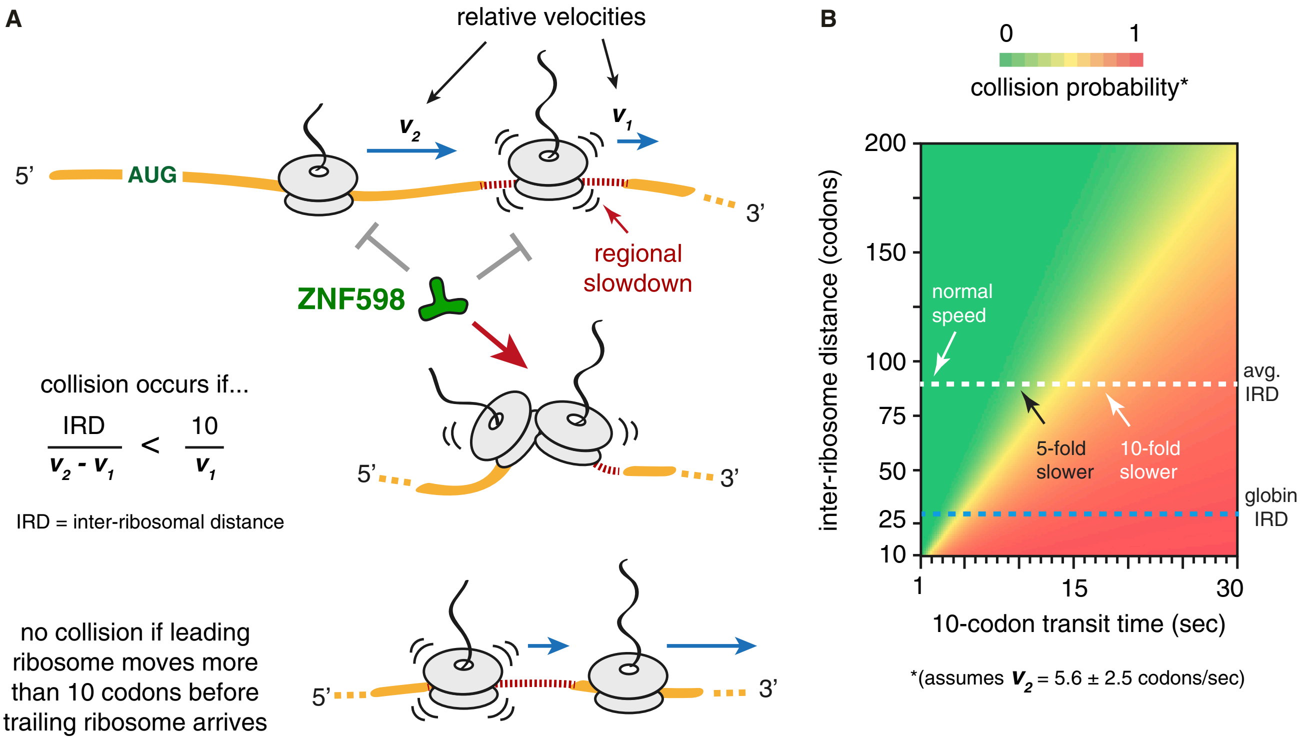

Figure 7. Model for Recognition of Aberrant Translation by ZNF598

(A) ZNF598 does not engage ribosomes that are translating normally or slow down at a difficult-to-translate sequence (regional slowdown). If the relative velocities of the lead and trailing ribosomes permit the trailing ribosome to close the inter-ribosomal distance (IRD) before the slowed ribosome moves 10 codons away, a collision will occur to allow recognition by ZNF598.

(B) Heatmap of the collision probability for a given slowdown (x axis) as a function of the distance to the trailing ribosome (y axis). An average velocity for the trailing ribosome of 5.6 ± 2.5 codons per second was used. At the genome-wide average IRD of 66 codons (~200 nt), appreciable collisions will not occur unless the lead ribosome slows to ~1 codon per second (less than one-fifth the normal rate) over a 10-codon stretch. However, for very frequently initiated mRNAs that have a very short IRD (such as globin mRNA), a mere 2-fold slowdown is sufficient to begin observing collisions.

terms. More importantly, the mechanism by which the ubiquitin mark(s) added by ZNF598 prevent further elongation and initiate downstream pathways of quality control and ribosome recycling remains to be investigated. Although the ASCC3 helicase (Slh1 in yeast) and associated factors have been functionally implicated in these steps (Matsuo et al., 2017; Sitron et al., 2017), their role is not known. The ability to now generate a defined ubiquitinated di-ribosome should provide a starting point for dissecting how this complex is subsequently disassembled, a critical step for both ribosome recycling and quality control.

Ribosome collisions on stall-containing mRNAs were recently shown to be required for endonucleolytic cleavage of those mRNAs in yeast (Simms et al., 2017). This suggests that both mRNA decay and nascent protein quality control rely on collisions. Whether both processes utilize the same sensor and effectors downstream of collisions remains to be established. The simplest model is that collisions recruit Hel2 and ubiquitinate key 40S residues. The ubiquitin would serve as a mark for factors that carry out mRNA cleavage, just as it is used to induce translation arrest, ribosome recycling, and nascent protein degradation (Garzia et al., 2017; Juszkiewicz and Hegde, 2017; Matsuo et al., 2017; Sundaramoorthy et al., 2017). Alternatively, the collided di-ribosome might recruit factors independently of Hel2 to initiate mRNA decay. In this light, it is noteworthy that endonucleolytic cleavage occurs at a position relative to the stalled ribosome (Guydosh and Green, 2017) that would correspond to interface 1. It will be important to determine whether Hel2 or 40S ubiquitination is a prerequisite for endonucleolytic cleavage and mRNA decay, and if not, what other collision-specific factor(s) are needed to execute these events.

Interaction of Hel2 with ribosomes in yeast may occur somewhat differently than ZNF598 in mammals. Hel2's seemingly constitutive ribosome association and ability to interact with monosomes (Matsuo et al., 2017) might indicate relatively stable binding near Asc1 (the RACK1 homolog), from where it could ubiquitinate uS10 only when a trailing ribosome collides. This model would reconcile constitutive Hel2-ribosome association with data suggesting that uS3 ubiquitination (which is near uS10) is enhanced by manipulations expected to cause ribosome collisions (Simms et al., 2017).

Because aberrant translation is detected via collisions, a cell's definition of "aberrant" is necessarily context and substrate specific. The P sites in a collided di-ribosome are separated by ~10 codons. Under normal conditions, the average inter-ribosomal distance between P sites is ~66 codons (Ingolia et al., 2011). At an elongation rate of ~5.6 codons per second (Ingolia

et al., 2011), a trailing ribosome will reach the position of the leading ribosome in 11.6 s. To avoid a collision, the leading ribosome must elongate at least 11 codons in this time. Thus, using transcriptome-wide averages, aberrant translation can now be defined as any 10-codon stretch where average elongation is slower than ~1 codon/s or roughly 5–6 times slower than normal. Stated more generally, collisions occur whenever a ribosome cannot elongate more than 10 codons before the trailing ribosome elongates the inter-ribosomal distance (Figure 7A).

Initiation frequency, inter-ribosomal distance, and speed of translation are influenced by mRNA properties, environmental stress, differentiation, and different physiologic states. Thus, the degree of slowdown needed to incur a ribosomal collision will differ for different mRNAs and be modulated by cellular conditions. For example, an infrequently translated mRNA can afford to pause for an extended period before a collision is incurred. By contrast, an extremely highly translated mRNA with a short inter-ribosomal distance would incur collisions with even transient elongation pauses (Figure 7B). A lower threshold for detecting stalls on highly translated mRNAs may benefit the cell because even a few aberrant copies of such mRNAs would generate aberrant proteins at a very high rate. Avoiding this risk may be more important than occasionally flagging normal stochastic collisions.

Among the most highly translated mRNAs in mammals are the globins in reticulocytes. Their ~140-codon open reading frames with 4–6 ribosomes (Figure S2B) will have inter-ribosomal distances of 20–40 codons, markedly increasing the risk of collisions during even transient slowdowns (Figure 7B). This appears to be tolerated by essentially eliminating ZNF598 in this cell type (Figure S1A), thereby forsaking quality control in favor of the massive translation rates needed to populate the cell with over 200 g/L hemoglobin. Thus, native polysomes in reticulocytes naturally have some stochastically collided di-ribosomes, a very fortuitous feature that in hindsight explains why nuclease-digested ribosomes from this source could be used to identify ZNF598 targets (Juszkiewicz and Hegde, 2017).

Earlier studies have shown that *in vivo* and *in vitro* polysomes can acquire a wide range of ribosome arrangements including beads-on-a-string, circles, spirals, and various closely packed configurations of uncertain biological relevance (Afonina et al., 2014; Brandt et al., 2009, 2010; Christensen and Bourne, 1999; Myasnikov et al., 2014). The collided ribosome characterized here matches one such configuration in which ribosomes form a helix (Myasnikov et al., 2014). Although this earlier structure was ascribed as actively translating polysomes, we can now infer that the super-helical polyribosome complex probably represents a stalled ribosome with a series of collided ribosomes behind it. Consistent with this interpretation, helical poly-ribosomes show reduced translation activity and are preferentially observed at later translation times (Afonina et al., 2015) when mRNA damage might induce stalling or the disassembly pathway has lost activity. Other ribosome configurations observed previously (Afonina et al., 2014; Brandt et al., 2009, 2010; Christensen and Bourne, 1999) might recruit (or be induced by) different factors and have functions other than quality control.

Ribosome collisions are also incurred in *E. coli* where they have been suggested to stimulate abortive termination of the leading ribosome by an unknown mechanism (Ferrin and Subramaniam, 2017). It will therefore be interesting to determine whether the prokaryotic collided ribosome has a distinctive architecture as hinted in earlier EM studies (Brandt et al., 2009). If so, factors might have evolved to recognize such a complex and induce abortive termination or signal other downstream events.

Linking specific polysome architectures with defined functions will afford the opportunity to use template matching (Mahamid et al., 2016) to directly visualize where in a cell specific translation-related events are occurring. Thus, the map of a collided di-ribosome presented here now provides a means to determine when, where, and how frequently ribosome-associated quality control is initiated in cells. More broadly, our findings reveal the general concept that differences in higher-order polysome structure can contain information that cellular factors exploit to modulate translation.

STAR★METHODS

Detailed methods are provided in the online version of this paper and include the following:

- KEY RESOURCES TABLE
- CONTACT FOR REAGENT AND RESOURCE SHARING
- EXPERIMENTAL MODEL AND SUBJECT DETAILS
  - Cell Lines
- METHOD DETAILS
  - Constructs
  - Cell Cultures
  - Flow Cytometry Analysis
  - *In Vitro* Transcription and Translation
  - Affinity Purification from RRL Translation Reactions
  - Denaturing His-Ub Pull-downs
  - Analytical Sucrose Gradient Fractionation
  - Western Blot Analysis
  - Analysis of Ribosome-ZNF598 Interaction in Cells
  - Recombinant ZNF598 Purification
  - Recombinant eRF1^{AAQ} Purification
  - Ribosome Purification and Ubiquitination Analysis
  - Analysis of Nuclease-Resistant Polysomes in Cells
  - Purification of Stalled Poly-ribosomes for Cryo-EM
  - Cryo-EM Grid Preparation
  - Data Collection
  - Data Processing
  - Collided Di-ribosome
  - Stalled Ribosome
  - Collided Ribosome
  - Principal Component Analysis
  - Model Building
  - Model Refinement and Validation
- QUANTIFICATION AND STATISTICAL ANALYSIS
- DATA AND SOFTWARE AVAILABILITY

SUPPLEMENTAL INFORMATION

Supplemental Information includes seven figures and one table and can be found with this article online at https://doi.org/10.1016/j.molcel.2018.08.037.

ACKNOWLEDGMENTS

We thank J. Grimmett, T. Darling, M. Daly, S. Chen, and C. Savva for technical support; J. Llacer, P. Emsley, G. Murshudov, and A. Brown for help with model building; Sjors Scheres and Takanori Nakane for help with multi-body refinement; and Hegde and Ramakrishnan lab members for useful discussions. This work was supported by the UK Medical Research Council (MC_UP_A022_1007 to R.S.H. and MC_U105184332 to V.R.), a Wellcome Trust Senior Investigator award (WT096570), the Agouron Institute, and the Louis-Jeantet Foundation (V.R.).

AUTHOR CONTRIBUTIONS

S.J. performed the majority of biochemical and cell biological experiments. V.C. developed methods to analyze polysomes by cryo-EM and determined the collided di-ribosome structure. Z.L. developed and used the nuclease-resistance assay to analyze collisions in cells. S.K. helped with sample preparation and EM analysis. V.R. and R.S.H. oversaw the project and provided guidance. R.S.H. wrote the manuscript with input from all authors.

DECLARATION OF INTERESTS

The authors declare no competing interests.

Received: April 27, 2018
Revised: August 7, 2018
Accepted: August 22, 2018
Published: October 4, 2018

REFERENCES

Afonina, Z.A., Myasnikov, A.G., Shirokov, V.A., Klaholz, B.P., and Spirin, A.S. (2014). Formation of circular polyribosomes on eukaryotic mRNA without cap-structure and poly(A)-tail: a cryo electron tomography study. Nucleic Acids Res. *42*, 9461–9469.

Afonina, Z.A., Myasnikov, A.G., Shirokov, V.A., Klaholz, B.P., and Spirin, A.S. (2015). Conformation transitions of eukaryotic polyribosomes during multi-round translation. Nucleic Acids Res. *43*, 618–628.

Anger, A.M., Armache, J.-P., Berninghausen, O., Habeck, M., Subklewe, M., Wilson, D.N., and Beckmann, R. (2013). Structures of the human and Drosophila 80S ribosome. Nature *497*, 80–85.

Barbacid, M., Fresno, M., and Vazquez, D. (1975). Inhibitors of polypeptide elongation on yeast polysomes. J. Antibiot. *28*, 453–462.

Becker, T., Armache, J.-P., Jarasch, A., Anger, A.M., Villa, E., Sieber, H., Motaal, B.A., Mielke, T., Berninghausen, O., and Beckmann, R. (2011). Structure of the no-go mRNA decay complex Dom34-Hbs1 bound to a stalled 80S ribosome. Nat. Struct. Mol. Biol. *18*, 715–720.

Behrmann, E., Loerke, J., Budkevich, T.V., Yamamoto, K., Schmidt, A., Penczek, P.A., Vos, M.R., Bürger, J., Mielke, T., Scheerer, P., and Spahn, C.M. (2015). Structural snapshots of actively translating human ribosomes. Cell *161*, 845–857.

Bengtson, M.H., and Joazeiro, C.A. (2010). Role of a ribosome-associated E3 ubiquitin ligase in protein quality control. Nature *467*, 470–473.

Brandman, O., and Hegde, R.S. (2016). Ribosome-associated protein quality control. Nat. Struct. Mol. Biol. *23*, 7–15.

Brandman, O., Stewart-Ornstein, J., Wong, D., Larson, A., Williams, C.C., Li, G.-W., Zhou, S., King, D., Shen, P.S., Weibezahn, J., et al. (2012). A ribosome-bound quality control complex triggers degradation of nascent peptides and signals translation stress. Cell *151*, 1042–1054.

Brandt, F., Etchells, S.A., Ortiz, J.O., Elcock, A.H., Hartl, F.U., and Baumeister, W. (2009). The native 3D organization of bacterial polysomes. Cell *136*, 261–271.

Brandt, F., Carlson, L.-A., Hartl, F.U., Baumeister, W., and Grünewald, K. (2010). The three-dimensional organization of polyribosomes in intact human cells. Mol. Cell *39*, 560–569.

Brown, A., Shao, S., Murray, J., Hegde, R.S., and Ramakrishnan, V. (2015a). Structural basis for stop codon recognition in eukaryotes. Nature *524*, 493–496.

Brown, A., Long, F., Nicholls, R.A., Toots, J., Emsley, P., and Murshudov, G. (2015b). Tools for macromolecular model building and refinement into electron cryo-microscopy reconstructions. Acta Crystallogr. D Biol. Crystallogr. *71*, 136–153.

Chakrabarti, O., and Hegde, R.S. (2009). Functional depletion of mahogunin by cytosolically exposed prion protein contributes to neurodegeneration. Cell *137*, 1136–1147.

Chen, V.B., Arendall, W.B., Headd, J.J., Keedy, D.A., Immormino, R.M., Kapral, G.J., Murray, L.W., Richardson, J.S., and Richardson, D.C. (2010). *MolProbity* : all-atom structure validation for macromolecular crystallography. Acta Crystallogr. Sect. D Biol. Crystallogr. *66*, 12–21.

Choe, Y.-J., Park, S.-H., Hassemer, T., Körner, R., Vincenz-Donnelly, L., Hayer-Hartl, M., and Hartl, F.U. (2016). Failure of RQC machinery causes protein aggregation and proteotoxic stress. Nature *531*, 191–195.

Christensen, A.K., and Bourne, C.M. (1999). Shape of large bound polysomes in cultured fibroblasts and thyroid epithelial cells. Anat. Rec. *255*, 116–129.

Chu, J., Hong, N.A., Masuda, C.A., Jenkins, B.V., Nelms, K.A., Goodnow, C.C., Glynne, R.J., Wu, H., Masliah, E., Joazeiro, C.A.P., and Kay, S.A. (2009). A mouse forward genetics screen identifies LISTERIN as an E3 ubiquitin ligase involved in neurodegeneration. Proc. Natl. Acad. Sci. USA *106*, 2097–2103.

Dimitrova, L.N., Kuroha, K., Tatematsu, T., and Inada, T. (2009). Nascent peptide-dependent translation arrest leads to Not4p-mediated protein degradation by the proteasome. J. Biol. Chem. *284*, 10343–10352.

Ferrin, M.A., and Subramaniam, A.R. (2017). Kinetic modeling predicts a stimulatory role for ribosome collisions at elongation stall sites in bacteria. eLife *6*, 6.

Frischmeyer, P.A., Van Hoof, A., O'Donnell, K., Guerrerio, A.L., Parker, R., and Dietz, H.C. (2002). An mRNA surveillance mechanism that eliminates transcripts lacking termination codons. Science *295*, 2258–2261.

Garzia, A., Jafarnejad, S.M., Meyer, C., Chapat, C., Gogakos, T., Morozov, P., Amiri, M., Shapiro, M., Molina, H., Tuschl, T., and Sonenberg, N. (2017). The E3 ubiquitin ligase and RNA-binding protein ZNF598 orchestrates ribosome quality control of premature polyadenylated mRNAs. Nat. Commun. *8*, 16056.

Grollman, A.P. (1968). Inhibitors of protein biosynthesis. V. Effects of emetine on protein and nucleic acid biosynthesis in HeLa cells. J. Biol. Chem. *243*, 4089–4094.

Guydosh, N.R., and Green, R. (2014). Dom34 rescues ribosomes in 3′ untranslated regions. Cell *156*, 950–962.

Guydosh, N.R., and Green, R. (2017). Translation of poly(A) tails leads to precise mRNA cleavage. RNA *23*, 749–761.

Higgins, R., Gendron, J.M., Rising, L., Mak, R., Webb, K., Kaiser, S.E., Zuzow, N., Riviere, P., Yang, B., Fenech, E., et al. (2015). The unfolded protein response triggers site-specific regulatory ubiquitylation of 40S ribosomal proteins. Mol. Cell *59*, 35–49.

Ingolia, N.T., Lareau, L.F., and Weissman, J.S. (2011). Ribosome profiling of mouse embryonic stem cells reveals the complexity and dynamics of mammalian proteomes. Cell *147*, 789–802.

Ishimura, R., Nagy, G., Dotu, I., Zhou, H., Yang, X.-L., Schimmel, P., Senju, S., Nishimura, Y., Chuang, J.H., and Ackerman, S.L. (2014). Ribosome stalling induced by mutation of a CNS-specific tRNA causes neurodegeneration. Science *345*, 455–459.

Ito-Harashima, S., Kuroha, K., Tatematsu, T., and Inada, T. (2007). Translation of the poly(A) tail plays crucial roles in nonstop mRNA surveillance via translation repression and protein destabilization by proteasome in yeast. Genes Dev. *21*, 519–524.

Izawa, T., Park, S.-H., Zhao, L., Hartl, F.U., and Neupert, W. (2017). Cytosolic protein Vms1 links ribosome quality control to mitochondrial and cellular homeostasis. Cell *171*, 890–903.e18.

Jiménez, A., Carrasco, L., and Vázquez, D. (1977). Enzymic and nonenzymic translocation by yeast polysomes. Site of action of a number of inhibitors. Biochemistry *16*, 4727–4730.

Juszkiewicz, S., and Hegde, R.S. (2017). Initiation of quality control during poly(A) translation requires site-specific ribosome ubiquitination. Mol. Cell *65*, 743–750.e4.

Kaida, D., Berg, M.G., Younis, I., Kasim, M., Singh, L.N., Wan, L., and Dreyfuss, G. (2010). U1 snRNP protects pre-mRNAs from premature cleavage and polyadenylation. Nature *468*, 664–668.

Koutmou, K.S., Schuller, A.P., Brunelle, J.L., Radhakrishnan, A., Djuranovic, S., and Green, R. (2015). Ribosomes slide on lysine-encoding homopolymeric A stretches. eLife *4*, 4.

Lareau, L.F., Hite, D.H., Hogan, G.J., and Brown, P.O. (2014). Distinct stages of the translation elongation cycle revealed by sequencing ribosome-protected mRNA fragments. eLife *3*, e01257.

Letzring, D.P., Wolf, A.S., Brule, C.E., and Grayhack, E.J. (2013). Translation of CGA codon repeats in yeast involves quality control components and ribosomal protein L1. RNA *19*, 1208–1217.

Lu, J., and Deutsch, C. (2008). Electrostatics in the ribosomal tunnel modulate chain elongation rates. J. Mol. Biol. *384*, 73–86.

Mahamid, J., Pfeffer, S., Schaffer, M., Villa, E., Danev, R., Cuellar, L.K., Förster, F., Hyman, A.A., Plitzko, J.M., and Baumeister, W. (2016). Visualizing the molecular sociology at the HeLa cell nuclear periphery. Science *351*, 969–972.

Marton, M.J., Vazquez de Aldana, C.R., Qiu, H., Chakraburtty, K., and Hinnebusch, A.G. (1997). Evidence that GCN1 and GCN20, translational regulators of GCN4, function on elongating ribosomes in activation of eIF2alpha kinase GCN2. Mol. Cell. Biol. *17*, 4474–4489.

Matsuo, Y., Ikeuchi, K., Saeki, Y., Iwasaki, S., Schmidt, C., Udagawa, T., Sato, F., Tsuchiya, H., Becker, T., Tanaka, K., et al. (2017). Ubiquitination of stalled ribosome triggers ribosome-associated quality control. Nat. Commun. *8*, 159.

Murshudov, G.N., Skubák, P., Lebedev, A.A., Pannu, N.S., Steiner, R.A., Nicholls, R.A., Winn, M.D., Long, F., and Vagin, A.A. (2011). REFMAC5 for the refinement of macromolecular crystal structures. Acta Crystallogr. D Biol. Crystallogr. *67*, 355–367.

Myasnikov, A.G., Afonina, Z.A., Ménétret, J.-F., Shirokov, V.A., Spirin, A.S., and Klaholz, B.P. (2014). The molecular structure of the left-handed supra-molecular helix of eukaryotic polyribosomes. Nat. Commun. *5*, 5294.

Nakane, T., Kimanius, D., Lindahl, E., and Scheres, S.H.W. (2018). Characterisation of molecular motions in cryo-EM single-particle data by multi-body refinement in RELION. eLife *7*, 286856.

Nicholls, R.A., Long, F., and Murshudov, G.N. (2012). Low-resolution refinement tools in REFMAC5. Acta Crystallogr. D Biol. Crystallogr. *68*, 404–417.

Pettersen, E.F., Goddard, T.D., Huang, C.C., Couch, G.S., Greenblatt, D.M., Meng, E.C., and Ferrin, T.E. (2004). UCSF Chimera–a visualization system for exploratory research and analysis. J. Comput. Chem. *25*, 1605–1612.

Pisareva, V.P., Skabkin, M.A., Hellen, C.U.T., Pestova, T.V., and Pisarev, A.V. (2011). Dissociation by Pelota, Hbs1 and ABCE1 of mammalian vacant 80S ribosomes and stalled elongation complexes. EMBO J. *30*, 1804–1817.

Rosenthal, P.B., and Henderson, R. (2003). Optimal determination of particle orientation, absolute hand, and contrast loss in single-particle electron cryomicroscopy. J. Mol. Biol. *333*, 721–745.

Saito, K., Horikawa, W., and Ito, K. (2015). Inhibiting K63 polyubiquitination abolishes no-go type stalled translation surveillance in Saccharomyces cerevisiae. PLoS Genet. *11*, e1005197.

Scheres, S.H.W. (2012). RELION: implementation of a Bayesian approach to cryo-EM structure determination. J. Struct. Biol. *180*, 519–530.

Shao, S., von der Malsburg, K., and Hegde, R.S. (2013). Listerin-dependent nascent protein ubiquitination relies on ribosome subunit dissociation. Mol. Cell *50*, 637–648.

Shao, S., Murray, J., Brown, A., Taunton, J., Ramakrishnan, V., and Hegde, R.S. (2016). Decoding mammalian ribosome-mRNA states by translational GTPase complexes. Cell *167*, 1229–1240.e15.

Sharma, A., Mariappan, M., Appathurai, S., and Hegde, R.S. (2010). In vitro dissection of protein translocation into the mammalian endoplasmic reticulum. Methods Mol. Biol. *619*, 339–363.

Shoemaker, C.J., and Green, R. (2012). Translation drives mRNA quality control. Nat. Struct. Mol. Biol. *19*, 594–601.

Shoemaker, C.J., Eyler, D.E., and Green, R. (2010). Dom34:Hbs1 promotes subunit dissociation and peptidyl-tRNA drop-off to initiate no-go decay. Science *330*, 369–372.

Simms, C.L., Yan, L.L., and Zaher, H.S. (2017). Ribosome collision is critical for quality control during no-go decay. Mol. Cell *68*, 361–373.e5.

Sitron, C.S., Park, J.H., and Brandman, O. (2017). Asc1, Hel2, and Slh1 couple translation arrest to nascent chain degradation. RNA *23*, 798–810.

Sundaramoorthy, E., Leonard, M., Mak, R., Liao, J., Fulzele, A., and Bennett, E.J. (2017). ZNF598 and RACK1 regulate mammalian ribosome-associated quality control function by mediating regulatory 40S ribosomal ubiquitylation. Mol. Cell *65*, 751–760.e4.

Tsuboi, T., Kuroha, K., Kudo, K., Makino, S., Inoue, E., Kashima, I., and Inada, T. (2012). Dom34:hbs1 plays a general role in quality-control systems by dissociation of a stalled ribosome at the 3′ end of aberrant mRNA. Mol. Cell *46*, 518–529.

van Hoof, A., Frischmeyer, P.A., Dietz, H.C., and Parker, R. (2002). Exosome-mediated recognition and degradation of mRNAs lacking a termination codon. Science *295*, 2262–2264.

Voorhees, R.M., Fernández, I.S., Scheres, S.H.W., and Hegde, R.S. (2014). Structure of the mammalian ribosome-Sec61 complex to 3.4 Å resolution. Cell *157*, 1632–1643.

Yonashiro, R., Tahara, E.B., Bengtson, M.H., Khokhrina, M., Lorenz, H., Chen, K.C., Kigoshi-Tansho, Y., Savas, J.N., Yates, J.R., Kay, S.A., et al. (2016). The Rqc2/Tae2 subunit of the ribosome-associated quality control (RQC) complex marks ribosome-stalled nascent polypeptide chains for aggregation. eLife *5*, e11794.

Zhang, K. (2016). Gctf: Real-time CTF determination and correction. J. Struct. Biol. *193*, 1–12.

Zheng, S.Q., Palovcak, E., Armache, J.-P., Verba, K.A., Cheng, Y., and Agard, D.A. (2017). MotionCor2: anisotropic correction of beam-induced motion for improved cryo-electron microscopy. Nat. Methods *14*, 331–332.

STAR★METHODS

KEY RESOURCES TABLE

REAGENT or RESOURCE	SOURCE	IDENTIFIER
Antibodies		
Rabbit polyclonal anti-ZNF598	Abcam	Cat. #ab80458; RRID: AB_2221273
Rabbit monoclonal anti-eS10	Abcam	Cat. #ab151550; RRID: AB_2714147
Rabbit polyclonal anti-uS5	Bethyl Labs	Cat. #A303-794A; RRID: AB_11218192
Rabbit monoclonal anti-uL2	Abcam	Cat. #ab169538; RRID: AB_2714187
Rabbit monoclonal anti-eS24	Abcam	Cat. #ab196652: RRID: AB_2714188
Mouse monoclonal anti-Flag	Sigma-Aldrich	Cat. #F3165; RRID: AB_259529
Rabbit polyclonal anti-GFP	Chakrabarti and Hegde, 2009	N/A
Rabbit polyclonal anti-RFP	Chakrabarti and Hegde, 2009	N/A
HRP conjugated mouse monoclonal anti-beta-Actin	Sigma-Aldrich	Cat. #A3854; RRID: AB_262011
HRP conjugated goat anti-rabbit	Jackson ImmunoResearch	Cat. #111-035-003 RRID: AB_2313567
HRP conjugated goat anti-mouse	Jackson ImmunoResearch	Cat. #115-035-003; RRID: AB_10015289
Chemicals, Peptides, and Recombinant Proteins		
3xFlag peptide	Sigma-Aldrich	Cat. #F4799
D-Biotin	Sigma-Aldrich	Cat. #47868<br>CAS: 58-85-5
Anti-Flag M2 affinity resin	Sigma-Aldrich	Cat. #A2220
Ni-NTA agarose	QIAGEN	Cat. #30210
Strep-Tactin Sepharose High-Performance	GE Healthcare	Cat. #28-9355-99
S7 Micrococcal Nuclease	Roche	Cat. #10107921001
Complete EDTA-free protease inhibitor cocktail	Roche	Cat. #11873580001
Pactamycin	Gift from E. Steinbrecher, Pharmacia Corp.	N/A
Cycloheximide	Sigma-Aldrich	Cat. #C4859;<br>CAS: 66-81-9
Hygromycin B	Millipore	Cat. #400051-100KU CAS: 31282-04-9
Blasticidin S	Santa Cruz Biotechnology	Cat. #sc204655 CAS: 3513-03-9
Didemnin B	Shao et al., 2016	N/A
Emetine	Calbiochem	Cat #324693; CAS #316-42-7
Anisomycin	Sigma-Aldrich	Cat. #A9789; CAS #22862-76-6
Doxycycline	Sigma-Aldrich	Cat. #D9891; CAS: 24390-14-5
EasyTag L-[^{35}S]-Methionine	Perkin Elmer	Cat. #NEG709A005MC
CAP (diguanosine triphosphate cap)	New England Biolabs	Cat. #S1404L
RNasin	Promega	Cat. #N251
Amino acid kit	Sigma-Aldrich	Cat. #09416
SP6 Polymerase	New England Biolabs	Cat. #M0207L
Creatine phosphate	Roche	Cat. #621714
Creatine kinase	Roche	Cat. #127566
His-Ubiquitin	Boston Biochem	Cat. # U-530
HA-Ubiquitin	Boston Biochem	Cat. # U-110
ZNF598-TEV-3xFlag (human)	Juszkiewicz and Hegde, 2017	N/A
eRF1-AAQ (human)	Brown et al., 2015a	N/A
UbcH5a	Boston Biochem	Cat. # E2-616
GST-UBE1 (human)	Boston Biochem	Cat. # E-306

(*Continued on next page*)

Continued

REAGENT or RESOURCE	SOURCE	IDENTIFIER
Experimental Models: Cell Lines		
HEK293T	ATCC	CRL-3216
ZNF598 KO Flp-In T-REx 293 dox inducible GFP-P2A-$(K^{AAA})_{21}$-P2A-RFP	Juszkiewicz and Hegde, 2017	N/A
WT Flp-In T-REx 293 dox inducible GFP-P2A-$(K^{AAA})_{21}$-P2A-RFP	Juszkiewicz and Hegde, 2017	N/A
WT Flp-In T-REx 293 dox inducible GFP-P2A-$(K)_0$-P2A-RFP	Juszkiewicz and Hegde, 2017	N/A
E. coli BL21(DE3) pLysS	Thermo Fisher Scientific	C606003
Recombinant DNA		
pSP64-Twin-Strep-VHPβ-$K(^{AAA})_{20}$-3F4	This study	N/A
pcDNA3.1 ZNF598-TEV-3xFlag	Juszkiewicz and Hegde, 2017	N/A
pRSETA 6xHIS-TEV-eRF1(AAQ)	Brown et al., 2015a	N/A
Primer: SP64 5′ Fwd: TCATACACATACGATTTAGG	Sharma et al., 2010	N/A
Primer: SP64 Rev: CAATACGCAAACCGCCTC	Sharma et al., 2010	N/A
Primer: SP64 STOP-truncation Rev: CTATGACATG ATTACGAATTCCTATCCGGTTTTGAG CCCAGG	This study	N/A
Software and Algorithms		
FlowJo	FlowJo	https://www.flowjo.com/
GraphPad Prism	GraphPad Software	https://graphpad.com/
RELION 2	MRC-LMB	https://www2.mrc-lmb.cam.ac.uk/relion/index.php?title=Main_Page
UCSF Chimera	UCSF	https://www.cgl.ucsf.edu/chimera/
Pymol	Schrödinger	https://pymol.org/2/
Coot	MRC-LMB	https://www2.mrc-lmb.cam.ac.uk/personal/pemsley/coot/
Adobe Illustrator	Adobe	https://www.adobe.com/uk/creativecloud.html
Other		
SuperSignal West Pico Chemiluminescent substrate	Thermo Fisher Scientific	Cat. #34080
Rabbit Reticulocyte Lysate Mix	Sharma et al., 2010	N/A
DMEM, high glucose, GlutaMAX Supplement, pyruvate	Thermo Fisher	Cat. #10569010
Tetracycline-free Fetal Calf Serum (FCS)	BioSera	Cat. #FB-1001T/500
PonceauS Solution	Sigma-Aldrich	Cat. P-7170-1L; CAS: 6226-79-5
Qubit RNA HS Assay Kit	Thermo Fisher	Cat. #Q32852

CONTACT FOR REAGENT AND RESOURCE SHARING

Further information and requests for resources and reagents should be directed to and will be fulfilled by the Lead Contact Ramanujan S. Hegde (rhegde@mrc-lmb.cam.ac.uk).

EXPERIMENTAL MODEL AND SUBJECT DETAILS

Cell Lines

All cell lines were cultured in Dulbecco's Modified Eagle's Medium (DMEM) with 10% fetal calf serum (FCS). In cases where the cells contained a doxycycline-inducible reporter, tetracycline-free FCS was used. 15 μg/ml blasticidin and 100 μg/ml hygromycin were included for culturing HEK293 TRex Flp-in cells stably expressing reporter constructs. All cell lines used in this study (listed in the Key Resources Table) have been described and characterized previously (Juszkiewicz and Hegde, 2017).

METHOD DETAILS

Constructs

Constructs for *in vitro* translation in rabbit reticulocyte lysate (RRL) were sub-cloned into a pSP64-based vector. The constructs contained an epitope tag at the N terminus (either Twin-Strep or FLAG), the cytosolic domain of Sec61β containing the autonomously folding villin headpiece (VHP) domain inserted between residues 14 and 15 (Shao et al., 2013), twenty AAA codons, and a C-terminal 3F4 tag. The FLAG-tagged ZNF598 construct (pcDNA3.1-ZNF598-TEV-3xFLAG) for recombinant protein production in mammalian cells has been described (Juszkiewicz and Hegde, 2017). The construct for *E. coli* expression and purification of human eRF1^{AAQ} (pRSETA-6XHIS-TEV-eRF1^{AAQ}) has been described (Brown et al., 2015a).

Cell Cultures

Induction of reporter expression for stalling assays was for 24 h with 1 μg/ml of doxycycline. Treatment with pactamycin was for 22 h at between 4 to 40 nM as indicated in the figure legends. Where indicated, treatment with elongation inhibitors was for 15 min with the following concentrations: anisomycin low dose 0.19 μM or high dose 76 μM; emetine low dose 1.8 μM or high dose 360 μM; Didemnin B low dose 0.5 μM or high dose 100 μM.

Flow Cytometry Analysis

Analysis of stalling using flow cytometry was essentially as described previously (Juszkiewicz and Hegde, 2017). In brief, cells were trypsinized, sedimented (1000 rpm for 5 min), resuspended in 10% FCS in PBS and analyzed using a Beckton Dickinson LSR II instrument. Approximately 20,000 GFP positive events were collected for each sample. Data analysis was performed using FlowJo software.

In Vitro Transcription and Translation

In vitro transcription was performed with SP6 polymerase as described previously, and utilized PCR products as the template (Sharma et al., 2010). The transcription reactions were conducted with 5-20 ng/μl PCR product in 40 mM HEPES pH 7.4, 6 mM $MgCl_2$, 20 mM spermidine, 10 mM reduced glutathione, 0.5 mM ATP, 0.5 mM UTP, 0.5 mM CTP, 0.1 mM GTP, 0.5 mM CAP, 0.4-0.8 U/μl RNasin and 0.4 U/μl SP6 polymerase at 37°C. *In vitro* translation in RRL was as described previously in detail (Sharma et al., 2010). Two RRL systems were employed: native, non-nucleased RRL was used to manipulate translation complexes on endogenous mRNA (predominantly α and β globin). For translation of exogenously added transcripts, the RRL was pre-treated with micrococcal nuclease to digest endogenous mRNAs as described before. Translation reactions typically contained 33% by volume crude RRL, 0.5 μCi/μl ^{35}S-methionine, 20 mM HEPES, 10 mM KOH, 40 μg/ml creatine kinase, 20 μg/ml pig liver tRNA, 12 mM creatine phosphate, 1 mM ATP, 1 mM GTP, 50 mM KOAc, 2 mM $MgCl_2$, 1 mM reduced glutathione, 0.3 mM spermidine and 40 μM of each amino acid except methionine. In cases where an exogenous transcript was used, the transcription reaction was added to 5% by volume to the translation reaction without further purification. Translations were for 20-45 min at 32°C unless indicated otherwise in the individual figure legends.

Affinity Purification from RRL Translation Reactions

200 μl or 400 μL translation reactions supplemented with between 2.5 to 10 nM recombinant ZNF598-3XFLAG were carried out for 25 min at 32°C before transferring to ice for the remainder of the experiment. 15 μL of StrepTactin High-Performance Sepharose or 15 μL of packed anti-FLAG affinity resin was added directly to the reaction and incubated for 1.5 h with constant gentle mixing at 4°C. The resin was washed 5 times on ice with Ribosome Nascent Chain (RNC) buffer (50 mM HEPES pH 7.6, 100 mM KOAc, 5 mM $Mg(OAc)_2$). Elution was for 30 min with RNC buffer containing 50 mM D-biotin on ice (for the StrepTactin resin) or 0.2 mg/ml of 3xFLAG peptide at 22°C (for FLAG resin). The eluate was separated from the resin for further analysis by autoradiography, and/or immunoblotting.

Denaturing His-Ub Pull-downs

In experiments where affinity purification of ubiquitinated products was desired, the translation reaction was supplemented with His_6-Ubiquitin to 10 μM. For the pull-down, the reaction was adjusted to 100 mM Tris pH 8.0 and 1% SDS in a final volume of 100 μl. The samples were boiled for 5 min at 95°C, cooled to room temperature, then diluted with 900 μL of pull-down buffer (PBS, 0.5% Triton X-100, 250 mM NaCl, 20 mM imidazole). 10 μl of packed Ni-NTA (QIAGEN) resin was added to the samples. Pul-ldowns were for 1.5 h at 4°C with end-over-end mixing. The resin was washed four times with 1 mL each of pull-down buffer before elution with SDS-PAGE sample buffer supplemented with 50 mM EDTA.

Analytical Sucrose Gradient Fractionation

Analytical sucrose gradient fractionations were performed as described with minor modifications (Juszkiewicz and Hegde, 2017). In brief, 20 μL of the sample was layered atop 200 μL of manually prepared 10%–50% or 20%–50% sucrose gradients in RNC buffer. Centrifugation was for 20 min at 55,000 rpm at 4°C using a TLS-55 rotor (Beckman) with the slowest acceleration and deceleration settings. Eleven 20 μL fractions were successively collected from the top and used directly for western blot analysis.

For autoradiography of the translated nascent chains, the samples were first digested with 0.15 mg/ml RNase A for 30 min on ice to remove the associated tRNA.

Western Blot Analysis

Western Blotting was performed as previously described (Juszkiewicz and Hegde, 2017). In brief, cells were washed twice with ice cold PBS and lysed with 100 mM Tris pH 8.0 with 1% SDS followed by boiling and vortexing to shear genomic DNA. Tris-tricine SDS-PAGE was followed by tank transfer to 0.2 μm nitrocellulose membrane. Primary antibodies were incubated for 1 h at room temperature or 4°C for 12 to 16 h. Detection used HRP-conjugated secondary antibodies and SuperSignal West Pico Chemiluminescent substrate.

Analysis of Ribosome-ZNF598 Interaction in Cells

Two 10 cm plates of cells at 80% confluency were used for each condition. Cells were treated with low or high dose of emetine (or DMSO as a vehicle control) for 15 min and transferred to ice. After 2 washes with ice cold PBS, cells were scraped from the dish, pelleted for 5 min at 1000 rpm, and resuspended in 200 μL of RNC buffer with 40 U/ml of RNAsin inhibitor (Promega), 0.01% digitonin, 1x EDTA-free protease inhibitor cocktail and 1 mM DTT. After incubation on ice for 15 min, the cells were disrupted mechanically by passage through a pre-chilled 26G needle using a 1 mL syringe. Cellular debris was removed by centrifugation for 15 min at 15,000 x g in a table-top microcentrifuge. 150 μg of protein in the lysate was adjusted to 20 μL with RNC buffer, fractionated on a sucrose gradient, and analyzed by western blotting analysis as described above.

Recombinant ZNF598 Purification

ZNF598 was expressed and purified as described previously (Juszkiewicz and Hegde, 2017). HEK293T cells were transfected with the ZNF598-TEV-3xFLAG construct using TransIt 293 (Mirus) and cultured for 48-72 h. The cells were washed twice with ice cold PBS, collected by scraping, sedimented, and lysed in RNC buffer with 100 μg/ml digitonin, 1 mM DTT and 1x EDTA-free cOmplete protease inhibitor cocktail (Roche) for 20 min on ice. The lysate was clarified in a table-top microcentrifuge at 4°C for 10 min at 16,100 x g and incubated with anti-FLAG affinity resin (Sigma) for 1.5 h at 4°C with constant mixing. The resin was washed thrice with 1 mL of lysis buffer, thrice with 50 mM HEPES pH 7.6, 400 mM KOAc, 5 mM $Mg(OAc)_2$, 100 μg/ml digitonin and 1 mM DTT and three times with RNC buffer. Elutions were carried out with one resin volume of 0.2 mg/ml 3xFLAG peptide in RNC buffer at 25°C for 30 min. Two sequential elutions were combined.

Recombinant eRF1^{AAQ} Purification

eRF1^{AAQ} was purified as before (Brown et al., 2015a) with slight modifications. All steps were performed at 4°C unless otherwise specified. Briefly, *E. coli* BL21 (DE3) pLysS was transformed with pRSETA/His$_6$-TEV-eRF1^{AAQ} and cultured in 2 L of LB with antibiotics at 37°C until an OD of 0.6 was reached. The cells were then induced with 0.25 mM IPTG and harvested after 2 hr. The cell pellet was lysed by sonicating in 50 mL of binding buffer (50 mM TRIS, 300 mM NaCl, 1 mM EDTA, 5 mM imidazole, 5% glycerol, 1 mM TCEP, pH 8.0) supplemented with 1% Triton X-100, 0.25% Na deoxycholate, 1 mM PMSF, 1 tablet cOmplete EDTA-free protease inhibitor, 5 mM $MgSO_4$, 5 mM $CaCl_2$ and 10 mg DNase I. The lysate was clarified by centrifugation for 20 min at 43,150 x g and the supernatant was filtered using 0.45 μm syringe filters and incubated with 5 mL of equilibrated cOmplete Ni resin (Roche) for 1 hr with rocking. The beads were washed with 50 mL binding buffer, 250 mL binding buffer supplemented with an additional 300 mM NaCl, again with 300 mL of binding buffer and eluted into 5 × 10 mL fractions of binding buffer supplemented with 250 mM imidazole pH 8.0. The His$_6$ tag was cleaved using 1 mg of home-made SuperTEV protease during overnight dialysis of the eluate against 1 L of 50 mM HEPES, 150 mM KOAc, 5 mM $Mg(OAc)_2$, 0.5 mM EDTA, 1 mM TCEP, pH 7.4. The dialysate was incubated with 5 mL of equilibrated Ni resin for 45 min with rocking and the flowthrough containing >95% pure eRF1^{AAQ} was concentrated to 9.1 μM, flash-frozen and stored at −80°C. Typical yield was ~9 mg of pure protein per L of culture.

Ribosome Purification and Ubiquitination Analysis

Analytical purification of collided ribosomes for subsequent ubiquitination reactions was performed using 500 μL of *in vitro* translation reaction using non-nucleased RRL supplemented with eRF1^{AAQ} to 1 μM. The reaction proceeded for 20 min at 32°C. Where indicated, the sample was then subjected to nuclease digestion (or mock treatment without nuclease) as follows: $CaCl_2$ was added to 0.5 mM and S7 nuclease to 150 U/ml; after 10 min at 25°C, the reaction was quenched by addition of EGTA to 1 mM. Reactions mixtures were spun through 250 μL of 20% sucrose cushion in RNC buffer for 1 h at 100,000 rpm at 4°C in the TLA-120.2 rotor. Ribosome pellets were washed once with 200 μL and resuspended with 50 μL of RNC buffer. *In vitro* ubiquitination of ribosomes was as described previously (Juszkiewicz and Hegde, 2017). Reactions contained an energy regeneration system (1 mM ATP, 40 ng/ml Creatine Kinase, 10 mM Creatine phosphate), 100 nM rhGST-UBE1, 200 nM UbcH5a, 100 nM ribosomes and 2.5-10 nM recombinant ZNF598 in RNC buffer. Reaction mixtures without ZNF598 and ribosomes were pre-incubated for 15 min at 22°C to precharge the E2 with ubiquitin. ZNF598 and ribosomes were added and incubated for 1 h at 32°C.

Analysis of Nuclease-Resistant Polysomes in Cells

One 10 cm plate of cells at 80% confluency was used per condition. Cells were treated for 15 min with low or high dose of emetine (or an equivalent volume of DMSO as a vehicle control). To analyze recovery from emetine, cells treated with low dose emetine were

washed twice with emetine-free media, harvested immediately (no recovery) or incubated with media lacking emetine for 1 hr and transferred to ice. After 2 washes with ice cold PBS, cells were collected and spun for 5 min at 1200 rpm. Lysis was in 300 μL of buffer containing 20 mM HEPES, pH 7.4, 100 mM KOAc, 5 mM $Mg(OAc)_2$, 0.5% Triton X-100, 1 mM DTT, and 1X EDTA-free protease inhibitor cocktail (Roche) for 15 min on ice. The lysate was clarified by centrifugation for 10 min at 15,000 x g at 4°C in a tabletop microcentrifuge. Total RNA in the lysate was quantified using the Qubit RNA HS Assay Kit (Thermo Fisher). Lysate containing 90 μg of total RNA was adjusted to 1 mM $CaCl_2$ and 0.5 U of S7 nuclease per μg of RNA in a total reaction volume of 300 μL. Digestion proceeded for 40 min at 25°C before the reaction was terminated by adding 1.2 μl of 500 mM EGTA. The sample was layered onto a 12 ml 10%–50% sucrose gradient and subjected to ultracentrifugation at 40,000 rpm for 2 h at 4°C in an SW40 rotor (Beckman). A piston gradient fractionator system (Biocomp) was used to monitor UV absorbance at 254 nm across the gradient.

Purification of Stalled Poly-ribosomes for Cryo-EM

4 × 1 mL of non-nucleased RRL supplemented with 500 nM $eRF1^{AAQ}$ was allowed to translate at 32°C for 25 min. Ribosomes from each reaction were pelleted through a 2 mL cushion of 15% (w/v) sucrose in PS buffer (50 mM HEPES, 100 mM KOAc, 2 mM $Mg(OAc)_2$, 0.5 mM TCEP, pH 7.4) by centrifugation at 424,480 x g for 1 hr at 4°C in a TLA-100.3 rotor (Beckman). The pellets were resuspended by soaking and repeated pipetting in 800 μL PS buffer supplemented with 1 U/μl RNasin inhibitor and carefully loaded onto two 14 mL sucrose gradients (10%–50%) in PS buffer supplemented with 20 U/ml RNasin inhibitor (Promega). The gradients were centrifuged at 202,048 x g for 2 hr at 4°C in an SW 40 Ti rotor (Beckman) and fractionated at 30 s per fraction (~0.65 mL each) using a peristaltic pump setup connected to a UV aborbance reader at 260 nm (Gilson). Fractions corresponding to tetra-ribosomes were pooled, diluted to 3 mL in PS buffer and the ribosomes pelleted by centrifugation at 424,480 x g for 1 hr at 4°C in a TLA-100.3 rotor. The pellets were resuspended in 25 μL of PS buffer supplemented with 1 U/μl RNasin inhibitor, quantified by $UV_{260\ nm}$ absorbance and used immediately for cryo-EM or flash-frozen and stored at −80°C. Analysis of a specimen prepared in this way by a repeat sucrose gradient verified that it contained little or no monosomes.

Cryo-EM Grid Preparation

Freshly-purified $eRF1^{AAQ}$-stalled poly-ribosomes were diluted to an absorbance at 260 nm of 7 (~140 nM) in PS buffer and 3 μL of the sample was applied at 4°C at 100% relative humidity to glow-discharged Quantifoil R2/2 grids coated with a ~60 Å layer of amorphous carbon prepared in-house. After incubation for 30 s and blotting for 4 s, the grid was plunge-frozen into liquid ethane using a Vitrobot Mk III (FEI) and stored in liquid N_2.

Data Collection

Refer to Table S1 for data statistics. All micrograph movies were recorded on an FEI Falcon III camera in integration mode using a 300 kV Titan Krios microscope equipped with an X-FEG source and using EPU software (FEI). Two datasets containing 2624 movies (43 frames; 2.03 e^- $frame^{-1}$ $Å^{-2}$) and 2520 movies (47 frames; 1.55 e^- $frame^{-1}$ $Å^{-2}$) were collected with total exposures of 1.6 s, each at a magnification of 75,000, resulting in a pixel size of 1.07 Å.

Data Processing

Data processing (Figure S3B) was performed in RELION-2.1 unless specified (Scheres, 2012). Movies were aligned using 5 × 5 patches using MotionCorr2 (Zheng et al., 2017) with dose-weighting. Contrast transfer function (CTF) was estimated using Gctf (Zhang, 2016) with equiphase averaging enabled and micrographs with good CTF (and corresponding to a CTF figure of merit > 0.19) were selected for further processing. Micrographs from both datasets were combined at this point. 364,776 particles were picked using Gautomatch (Kai Zhang, MRC-LMB) and extracted in a 450 pixel box, which was then downscaled three-fold into a 150 pixel box (3.21 Å/pixel). Two rounds of two-dimensional classification were performed to yield 257,331 ribosome particles. Initial three-dimensional refinement was carried out using a 70 Å lowpass-filtered reference map of a rabbit ribosome (EMDB 3039) as initial model and generated a starting ribosome map.

Collided Di-ribosome

We first identified the subset of ribosomes stalled at the stop codon using focused classification with partial signal subtraction (FCwSS) as described previously (Brown et al., 2015a). Briefly, PDB 5LZV (Shao et al., 2016) was docked into the density of our starting ribosome map using UCSF Chimera and all ribosome signal outside a generous mask around eRF1 and ABCE1 was subtracted. Masked 3D classification into 4 classes was performed without image alignment for 25 iterations and a regularisation parameter (T) of 10. The procedure yielded a 28% class with unambiguous density for $eRF1^{AAQ}$-ABCE1. Visual inspection of the selected stalled ribosomes on raw micrographs revealed that some, but not all particles were part of closely packed poly-ribosomes. As monosomes were not present in appreciable amounts in the sample, we can infer that some degree of polysome dissociation occurred during vitrification. We therefore used a second FCwSS procedure to separate isolated stalled ribosomes from those that were part of higher-order di- and poly-ribosomes. Stalled ribosome particles were re-extracted and re-refined in a 1870 pixel box that was down-scaled to 374 pixels (5.35 Å/pixel). The resulting map had clear but poorly aligned density for a neighboring collided ribosome on the E-site side of the stalled ribosome. Importantly, no density was observed on the A-site side, providing an independent validation that these were indeed the leading stalled ribosome. Signal from the stalled ribosome was subtracted and the particles

were classified into 4 classes without image alignment inside a generous mask around the collided ribosome for 25 iterations (T = 10). Two classes containing density for the collided ribosome and together comprising 25% of the input data (14,634 particles) were selected, the subtracted signal was reverted and the data refined to a final angular accuracy of 1.3° and reached Nyquist resolution (10.8 Å).

Re-centering the di-ribosome to its approximate center of mass was necessary in order to minimize the box size, pixel size, and computational costs needed to reach a higher resolution. To this end, the center of the di-ribosome was manually repositioned in UCSF Chimera to coincide with the center of the box and a translation only search was performed in the same dataset over a range of 50 pixels in initial steps of 5 pixels. Finally, the re-centered di-ribosome particles were re-extracted in a 1000 pixel box, downscaled two-fold to 500 pixel (2.14 Å/pixel) and re-refined to an angular accuracy of 0.6°. The resulting map of the collided di-ribosome had an overall resolution of 6.1 Å.

The stated resolution of this initial di-ribosome map did not truly represent the observed map quality, which clearly suffered due to continuous structural heterogeneity in the relative orientations between the two ribosomes. Accurate alignment of the stalled ribosome smeared the density of the collided ribosome (Figure S4C), and vice versa. The recently described multi-body refinement (Nakane et al., 2018) was therefore performed to account for changes in the relative orientations of the two ribosomes. Using a pre-release version of RELION-3.0, each ribosome was treated as an independently moving rigid body and signal from each body was in turn subtracted on the fly and the partial signal subtraction itself was improved during each iteration of the focused refinement until convergence. Standard deviations of the rotation priors were 10° and 20°, respectively for the stalled and collided ribosomes, the corresponding standard deviations for the translation priors were 2 and 4 pixels, the initial angular sampling rate was 3.7° and the translation sampling rate was 0.75 pixel. The resulting collided di-ribosome map (Figure S4D) shows solvent-corrected resolution estimates for the stalled and collided ribosomes to be 6.8 Å and 6.5 Å, respectively. Regions of the collided ribosome farthest away from the center of mass of the di-ribosome, such as the L1 stalk became readily interpretable and helped to infer that the collided ribosome was in the rotated state with a ‘closed’ L1 stalk (compare Figure S4C versus Figure S4D).

The resulting collided di-ribosome map from the multi-body refinement contained each body at its refined center of mass and was used for docking of models derived from higher resolution maps of the stalled and collided ribosomes obtained from the same dataset as described below.

Stalled Ribosome

The 28% class of all ribosomal particles with unambiguous density for eRF1^{AAQ}-ABCE1 was subjected to 3D refinement to a final angular accuracy of 0.5°, movie refinement, particle polishing, another round of 3D refinement and post-processing using a generous solvent mask. This yielded a 3.9 Å map of the stalled ribosome (Figure S4A). The ribosomes that comprise this map were all part of polysomes in the sample, but only a subset remained in polysome complexes after vitrification. Nevertheless, comparison of this map to the stalled ribosome in the collided di-ribosome map above showed them to be the same with respect to rotation state, presence of factors, and positions of all key features. This suggests that the individual stalled ribosomes resulting from dissociated polysomes largely retained their conformation, allowing us to use this higher resolution map for building atomic models (see below), which could then be docked into the di-ribosome map.

Collided Ribosome

Three-dimensional classification of all ribosomal particles without alignments separated the particles broadly into canonical and rotated states (Figure S3B, top right), producing two major classes. The first major class contained canonical ribosomes with partial occupancy in the A- and F-sites for eRF1^{AAQ} and ABCE1, respectively (44%). This class presumably contains within it the subset of particles that produced the stalled ribosome map above, plus others from which the factors may have dissociated. The second major class contained rotated ribosomes with strong density for A/P and P/E hybrid tRNAs (38%). Particles in this class are necessarily not the lead stalled ribosome. Particles from this class were re-extracted in a 356 pixel box (1.52 Å/pixel; corresponding to a Nyquist resolution of 3.04 Å) and refined against their resampled, 70 Å lowpass-filtered map as initial reference to a final angular accuracy of 0.5°. The particles were then subjected to movie refinement, particle polishing, 3D refinement, and automated B-factor sharpening and masked FSC calculation (post-processing) using a featureless solvent mask to yield a 3.8 Å map (Figure S4B). The ribosomes that comprise this map were all part of polysomes in the sample, but only a subset remained in polysome complexes after vitrification. However, comparison of this map to the collided ribosome in the collided di-ribosome map above showed them to be the same with respect to rotation state, presence, and states of the tRNAs, and positions of all key features. This suggests that the individual collided ribosomes resulting from dissociated polysomes largely retained their conformation, allowing us to use this higher resolution map for building atomic models (see below), which could then be docked into the di-ribosome map.

Principal Component Analysis

The flex analyzer module of RELION-3.0 (pre-release) was used to quantify the inter-ribosomal motions seen in individual di-ribosome particles using principal component analysis. Briefly, motions in the data were described as linear combinations of principal components to account for variations in the data along independent degrees of freedom. The eigenvectors corresponding to each linear combination were rank-ordered by importance (Figure S4E) and the particles were distinguished based on the associated eigenvalue (Figure S4F). The particles were binned by eigenvalue into 10 bins, and maps representing the median orientations in each bin were

generated for each eigenvector. Eigenvector 1, which accounted for 42% of variance in the data, and which described pivoting of the collided ribosome about an axis along the length of the 40S was used to produce the heatmap of variance shown in Figure 4B. To produce this heatmap, the upper and lower 10% of eigenvector 1 particles (Figure S4F) from multi-body refinement and principal component analysis were used to produce two maps that represent the extremes in variation. Collided ribosome models were docked into these two maps, and the distance between equivalent atoms was computed and encoded as atomic B-factors in the PDB file for display of relative variance.

Model Building

Stalled Ribosome

PDB 5LZV (Shao et al., 2016) was docked into the sharpened density for the stalled ribosome and the E-site tRNA chain was deleted. The L1 and P stalk RNA and protein coordinates were taken from PDB 4V6X (Anger et al., 2013). The model was adjusted manually in Coot to conform with the density using suitably blurred maps (with B-factors between −50 and −150), real space-refined to improve the fit and 50 cycles of local refinement with a radius of 6 Å and a weight of 0.00001 was performed in Refmac v 5.8 (Murshudov et al., 2011) in the presence of ProSMART self-restraints (Nicholls et al., 2012) and LIBG base pairing and stacking restraints (Brown et al., 2015b). Poly-alanine was modeled into the nascent chain. The stop codons in rabbit α- and β-hemoglobin are UAA and UGA, respectively, and the latter was built into the density. The P-site tRNA model from 5LZV was retained. The P-site codons are CGU (α) and CAC (β), which code for Arg and His residues, respectively, at the protein C-termini. The wobble position was chosen to be cytosine and the corresponding tRNA base changed to guanine, the middle position was set to adenine (in lieu of the purines A/G) and the first position was set to cytosine (in lieu of the pyrimidines C/U). The mRNA from 5LZV was manually adjusted into a map blurred with an overall B-factor of −200. Finally, the three bundles comprising the model were combined using *phenix.combine_models* and refined for 12 macro-cycles using *phenix.real_space_refine*. Refer to Table S1 for statistics.

Collided Ribosome

The large and small subunits of PDB 5LZV were individually docked into sharpened density for the collided ribosome. The P and L1 stalks and associated proteins were adjusted manually to fit the density in the open and closed conformations, respectively. The A/P and P/E tRNAs were taken from the porcine rotated ribosome structure [PDB 3J7R (Voorhees et al., 2014)] and poly-alanine was used to model the nascent chain. The mRNA from 3J7R was extended from as described for the stalled ribosome and the P-site codon was tentatively set to Val138 of β-hemoglobin. Real and reciprocal space refinement was performed using Coot, REFMAC and phenix as above.

Collided Di-ribosome

The generated stalled and collided ribosome models were rigid body fitted into the collided di-ribosome map without any further adjustments. The structure of the interface (Figure 3) must therefore be interpreted as the median conformation of the interacting elements and the plasticity of the interface allows other related geometries described by the principal component analysis.

Model Refinement and Validation

Models were refined with REFMAC v5.8 utilizing external restraints generated by ProSMART and LIBG (Murshudov et al., 2011; Nicholls et al., 2012). Model statistics (Table S1) were obtained using MolProbity (Chen et al., 2010).

Molecular Graphics

Structural figures were generated using Pymol (Schrödinger, LLC; Figures 3 and 4B) or UCSF Chimera (Pettersen et al., 2004) (Figures 4A, S3, and S4). The FSC curves in Figures S4A, S4B, and S4D were generated by RELION 2.1 and and redrawn in Adobe Illustrator. The graphs in Figures S4E and S4F were generated by RELION 3.0 (pre-release) and redrawn in Adobe Illustrator.

QUANTIFICATION AND STATISTICAL ANALYSIS

All reported resolutions are based on the Fourier shell correlation (FSC) 0.143 criterion (Rosenthal and Henderson, 2003).

DATA AND SOFTWARE AVAILABILITY

Four maps have been deposited with the EMDB with accession codes EMD-0192 (stalled ribosome), EMD-0194 (collided ribosome), EMD-0195 (collided di-ribosome – stalled) and EMD-0197 (collided di-ribosome - collided). Atomic coordinates have been deposited with the Protein Data Bank under accession codes PDB 6HCF (stalled ribosome), 6HCJ (collided ribosome), 6HCM (collided di-ribosome - stalled) and 6HCQ (collided di-ribosome - collided).

Molecular Cell, Volume *72*

Supplemental Information

ZNF598 Is a Quality Control Sensor of Collided Ribosomes

Szymon Juszkiewicz, Viswanathan Chandrasekaran, Zhewang Lin, Sebastian Kraatz, V. Ramakrishnan, and Ramanujan S. Hegde

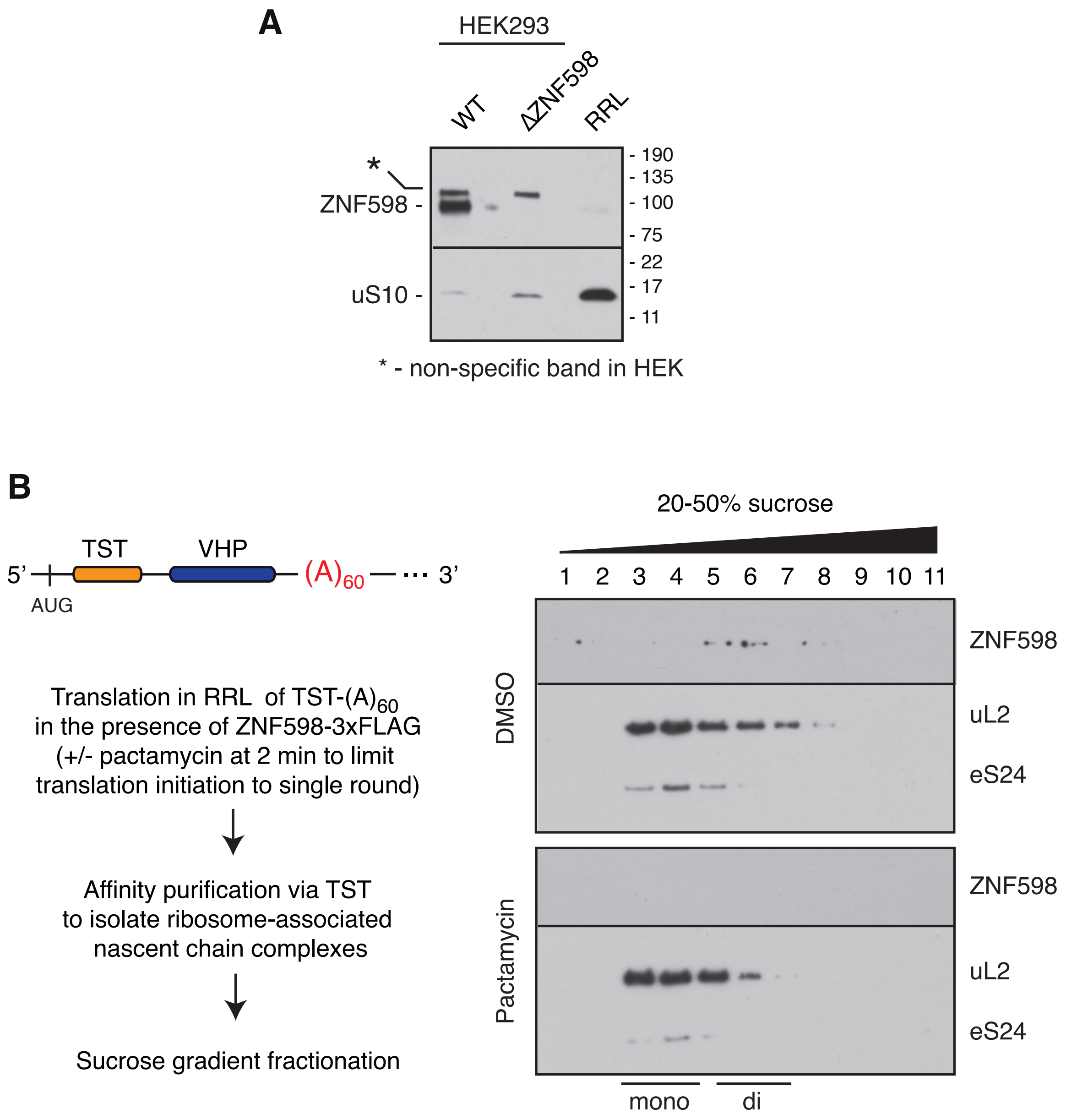

Fig. S1. ZNF598 engages a subpopulation of poly(A)-stalled ribosomes in vitro (related to Fig. 1)
(A) Lysates from WT or ΔZNF598 HEK293 cells were compared with rabbit reticulocyte lysate (RRL) by immunoblotting. Only a trace amount of ZNF598 was detected in RRL despite over-loading the sample (as judged by ribosomal protein uL10 levels). **(B)** Left panel: schematic representation of the TST-$(A)_{60}$ construct and experimental strategy to confine initiation in vitro to a single round by adding an initiation inhibitor (pactamycin) 2 min after the start of translation. Right panel: ribosome-nascent chain complexes from the mock treated (top) or pactamycin treated (bottom) translation reactions of TST-$(A)_{60}$ were affinity purified via the nascent chain. Equal amounts of recovered ribosomal complexes (as judged by absorbance at 260 nm) were separated by centrifugation through a sucrose gradient followed by immunoblotting of individual fractions for ZNF598 and ribosomal proteins. The positions of mono-ribosome and di-ribosome fractions are indicated. Note that recovery of ZNF598 with the ribosomal complexes is diminished when re-initiation is minimized. The reduced proportion of ribosomes in the di-ribosome fractions of the pactamycin-treated sample verifies that re-initiation was indeed reduced.

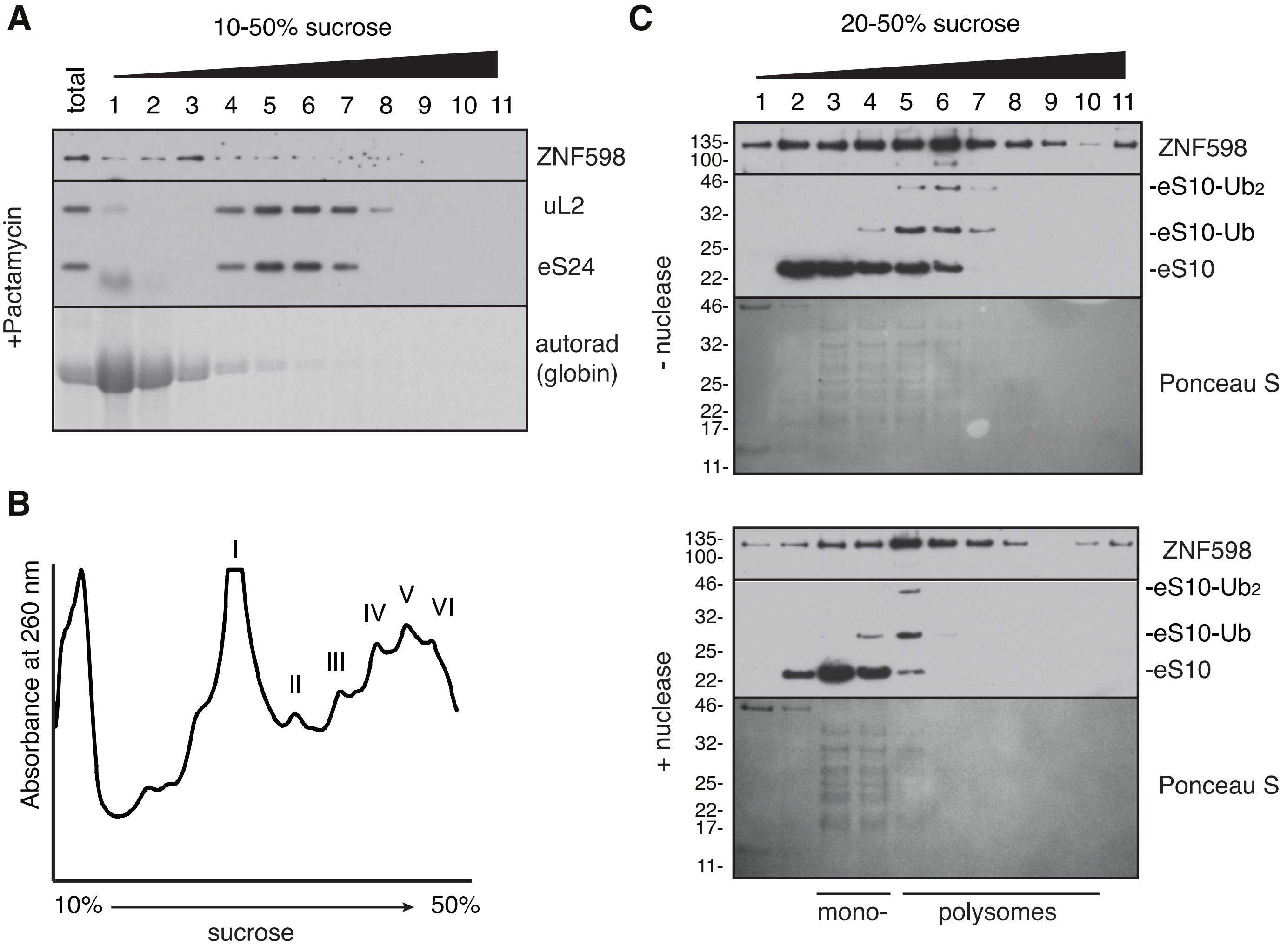

Fig. S2. Site-specific stalling of native polysomes triggers ZNF598 engagement (Related to Fig. 2)
(A) Native RRL was supplemented with 5 nM ZNF598 and the initiation inhibitor pactamycin before allowing ribosomes to run-off in the presence of ^{35}S-methionine. The reaction was analzyed on a sucrose gradient followed by autoradiography (to detect newly translated globin) or immunblotting for ZNF598 and ribosomal proteins. This reaction was performed and analyzed in parallel with that shown in Fig. 2B, which was stalled at the termination codon with eRF1^{AAQ}. Comparison of the two results indicates that the run-off sample produces full length globin that is not ribosome-associated, while the eRF1^{AAQ}-stalled sample contains an appreciable proportion of ribosome-associated globin. Furthermore, ZNF598 in the run-off sample is not stably associated with ribosomes, in contrast to the eRF1^{AAQ}-stalled sample. **(B)** Native RRL supplemented with eRF1^{AAQ} was allowed to translate for 10 min as in Fig. 2B before separation by sucrose gradient. The absorbance at 260 nm across the gradient is shown. Peak fractions corresponding to monosomes through pentasomes (I through V, respectively) were recovered for in vitro ubiquitination reactions with purified ZNF598 as shown on Figure 2D. The tetrasome fraction was also the sample used for subsequent structural analysis. **(C)** An experiment as Figure 2E is shown with the ubiquitinated and unmodified forms of eS10 detected simultaneously on the same strip of the blot. Note that after nuclease digestion, eS10 in the nuclease-resistant polysome fraction (lane 5) is almost completely ubiquitinated, whereas eS10 in the monosome fractions (lane 3) is almost entirely unmodified. ZNF598 is seen across the gradient due to over-exposure of the blot and some dissociation during the gradient centrifugation. Nevertheless, it is enriched in the fractions that also show the most eS10 ubiquitination.

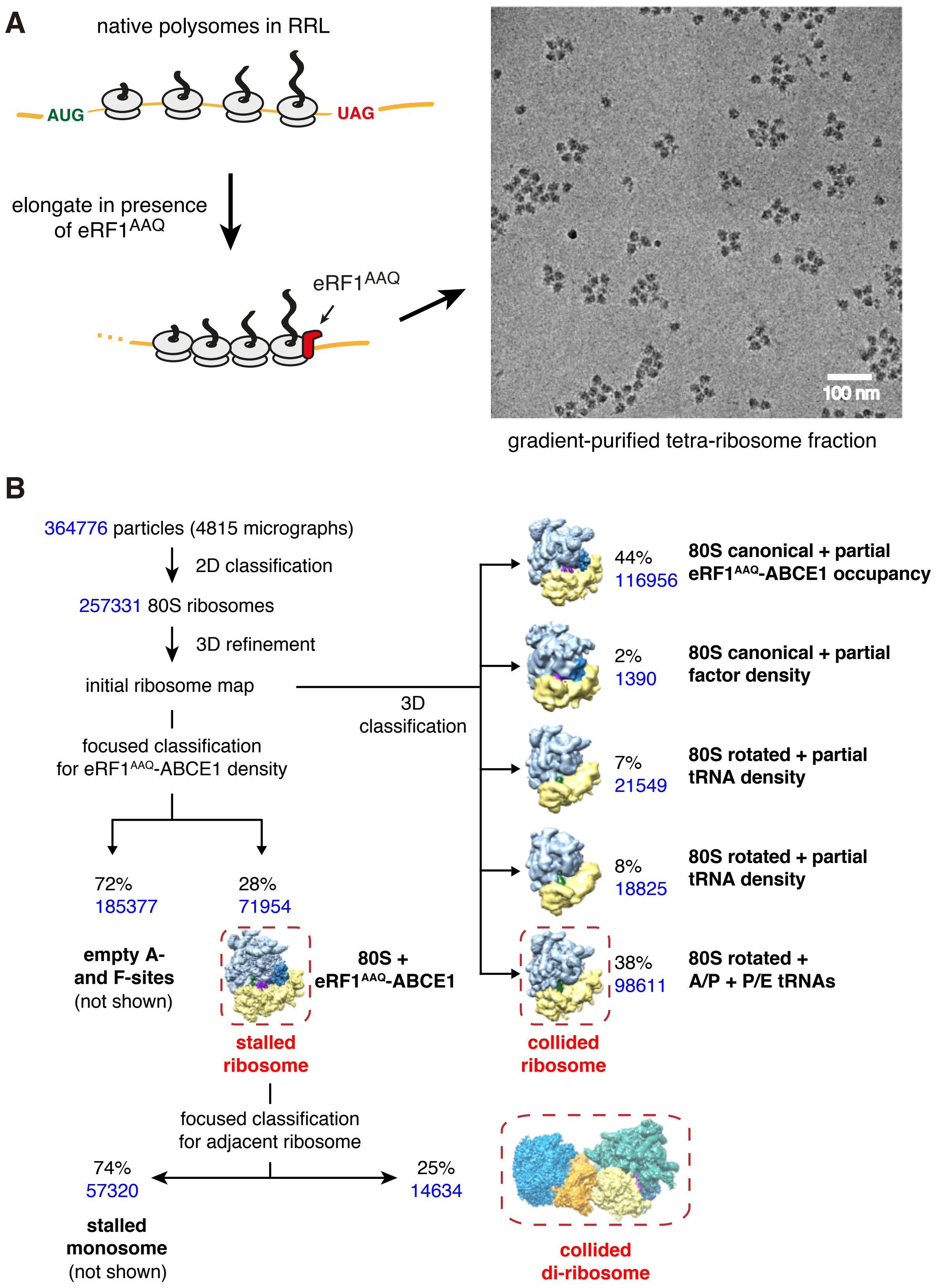

Fig. S3. Experimental strategy and classification scheme for structure determination (related to Fig. 3) **(A)** Schematic for generating native (primarily globin) stalled poly-ribosome complexes for cryo-EM analysis. Experimental details are provided in the Methods. The micrograph shows that although the tetra-ribosome fraction was analyzed, contamination with tri- and penta-ribosomes is readily seen. In addition, some disassembly during vitrification results in mono-ribosomes. **(B)** Overview of the classification scheme used to identify the leading stalled ribosomes and trailing collided ribosomes. Particles representing the stalled ribosome were used for additional classification to identify those that contain an adjacent collided ribosome, yielding a collided di-ribosome map. The particles represented in the red boxes were subjected to movie processing, particle polishing, 3D refinement, and in the case of the collided di-ribosome, multi-body refinement.

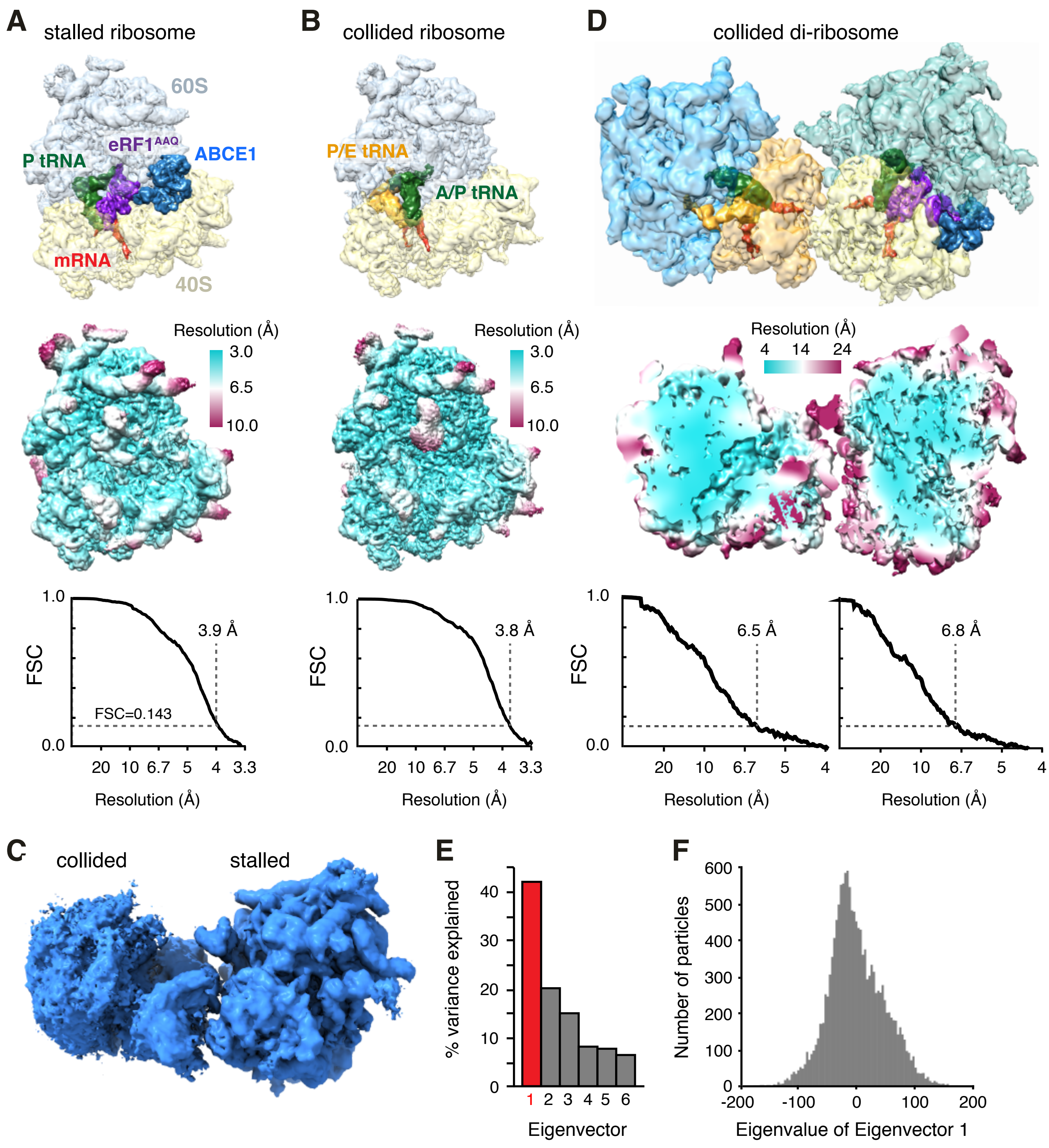

Fig. S4. Map Quality and Conformational Heterogeneity in Collided Di-ribosomes (related to Fig. 4). **(A, B)** Cryo-EM maps from this study (before post-processing) colored according to bound factors (top panels) and local resolution (middle panels). The bottom panels show Fourier shell correlation (FSC) curves computed between independent half-maps (black). The resolution estimated from map-to-map correlation at FSC = 0.143 is indicated. **(C)** Collided di-ribosome map with accurate alignment of the stalled ribosome. Notice how the rotation state, identity of bound factors and conformations of expansion segments of the collided ribosome are ambiguous due to structural heterogeneity. **(D)** The improved collided di-ribosome map resulting from multi-body refinement in which the stalled and collided ribosomes are treated independently. The middle panel shows local resolution through a section of the map, and the bottom panel shows FSC curves for the stalled and collided ribosomes in the map. **(E)** Histogram showing contributions of the top 6 eigenvectors from multi-body refinement and principal component analysis towards explaining structural variations among collided di-ribosome particles. **(F)** Histogram of number of particles versus eigenvalue for eigenvector 1 from panel E, which explains 42% of the variance in the structure of the collided di-ribosome.

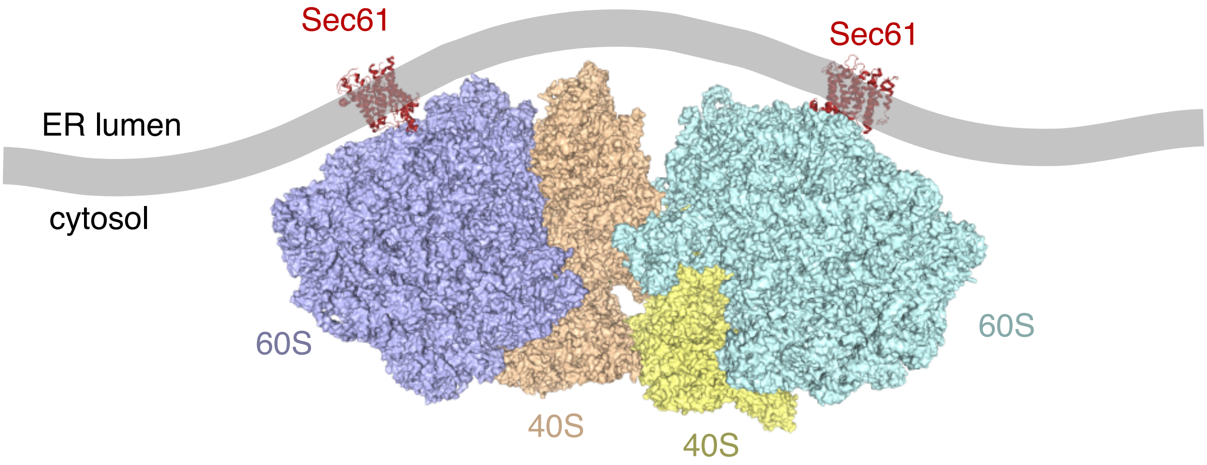

Fig. S5. Compatibility of the collided di-ribosome with membrane binding (related to Fig. 4).
A large proportion of total cellular translation occurs on ribosomes bound to the ER membrane. To determine whether the collided di-ribosome strucutre is compatible with membrane bound ribosomes, the Sec61 translocon (red) was docked into its binding site on each ribosome of the collided di-ribosome. The shaded bar depicts a hypothetical membrane bilayer, illustrating that it is compatible with the positions of Sec61 with some bending. This degree of membrane bending is easily within the range that is observed for ER membrane in electron micrographs. Furthermore, the flexibility of the di-ribosome at its interface (Fig. 4) may further facilitate collisions at the membrane surface.

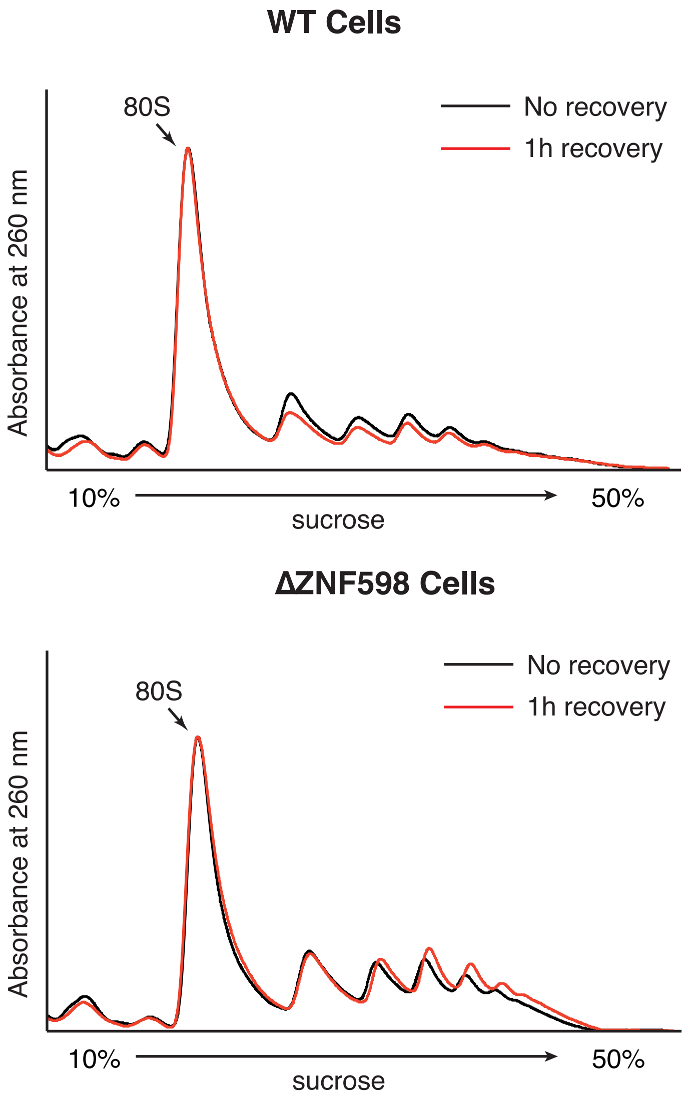

Fig. S6. Resolution of collided ribosomes relies on ZNF598 (related to Figure 5)
WT (top panel) or ΔZNF598 HEK293 cells were treated with 1.8 μM emetine for 15 minutes. One aliquot was harvested immediately, while another was allowed to recover for 1 hour in emetine-free medium before harvesting. Cytosol from each of the samples was digested with micrococcal nuclease and separated by sucrose gradient sedimentation. The absorbance at 260 nm across the gradient was plotted, normalized to the peak 80S absorbance. Note that the nuclease-resistant di-, tri-, and poly-ribosome peaks are partially resolved during the one hour recovery in WT cells, but persist in ΔZNF598 cells. In fact, the proportion of tetra-ribosomes and larger polysomes increases in ΔZNF598 cells, consistent with additional ribosome loading onto already stalled complexes.

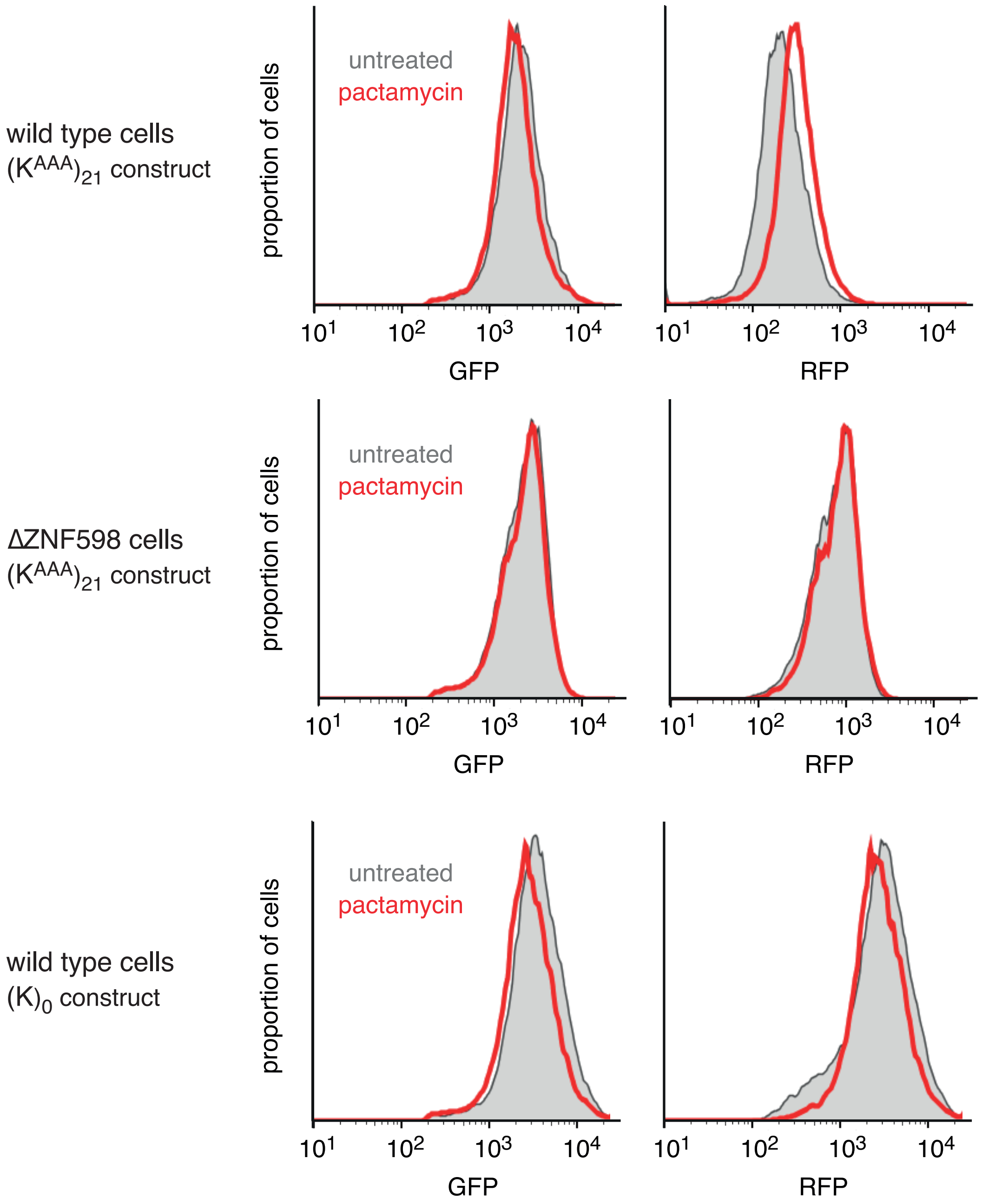

Fig. S7 Detection of ribosome stalling is context dependent (related to Figure 6)
Histograms for individual fluorescent protein signals from the indicated cells expressing the indicated constructs. The cells were either untreated or treated for 22 h with 10 nM pactamycin to partially inhibit initiation. Note that there is a selective increase in RFP expression only in wild type cells expressing $(K^{AAA})_{21}$ stalling reporter, which explains the observed increase in RFP:GFP ratio shown in Fig. 6B. Thus, the change in ratio can be ascribed to an increase in read-through of the poly(A) stalling sequence.

Table S1. Data collection, processing, refinement and model statistics (related to Figure 3).

	Stalled ribosome	Collided ribosome	Di-ribosome
**Data Collection**			
Voltage (kV)	300		-
Pixel size (Å)	1.07		-
Defocus range (µm)	-1.5 to -3.0		-
Defocus mean (µm)	-2.25		-
Electron dose (e^{-} $frame^{-1}$ $Å^{-2}$)	2.03, 1.55		
**Data Processing**			
Independent data collections	2		-
Useable micrographs	4815		-
Particle picking software	Gautomatch		-
Particles picked	364,776		-
Particles after 2D classification	257,331		-
Final particles	71,954	98,611	14,634
Map sharpening B-factor ($Å^2$)	-101.6	-86.3	-90.8(stalled)<br>-77.8(collided)
Resolution (Å)	3.9	3.8	6.8 (stalled)<br>6.5 (collided)
PDB accession code	6HCF	6HCJ	6HCM (stalled)<br>6HCQ (collided)
EMDB accession code	EMD-0192	EMD-0194	EMD-0195 (stalled)<br>EMD-0197 (collided)
**Refinement**			
Resolution (Å)	3.9	3.8	
Average B factor ($Å^2$)	75.4	96.7	
$FSC_{average}$	0.75	0.80	-
**R.M.S deviations**			
Bond lengths (Å)	0.019	0.013	-
Bond angles (°)	1.188	1.11	-
**Validation**			
Molprobity score	1.83	1.81	
Clashscore, all atoms (percentile)	5.27 (93rd)	4.53 (95th)	-
Favored rotamers (%)	94.9	95.0	-
Poor rotamers (%)	0.37	0.33	-
**Ramachandran plot**			
Favored (%)	90.0	88.9	-
Outliers (%)	0.2	0.34	-
**Validation (RNA)**			
Correct sugar puckers (%)	98.8	98.8	-
Good backbone conformations (%)	70	67	-
